# NADPH Oxidase 5 (NOX5) Upregulates MMP-10 Production and Cell Migration in Human Endothelial Cells

**DOI:** 10.3390/antiox13101199

**Published:** 2024-10-03

**Authors:** Javier Marqués, Elena Ainzúa, Josune Orbe, María Martínez-Azcona, José Martínez-González, Guillermo Zalba

**Affiliations:** 1 Navarra Institute for Health Research (IdiSNA), 31008 Pamplona, Spain; jmarquesc@unav.es (J.M.); eainzua@unav.es (E.A.); josuneor@unav.es (J.O.); mmazcona@unav.es (M.M.-A.); 2 Department of Biochemistry and Genetics, University of Navarra, 31009 Pamplona, Spain; 3 Atherothrombosis Laboratory, Cardiovascular Diseases Program, CIMA (University of Navarra), 31008 Pamplona, Spain; 4 RICORS-Ictus, Carlos III Health Institute, 28029 Madrid, Spain; 5 Instituto de Investigaciones Biomédicas de Barcelona-Consejo Superior de Investigaciones Científicas (IIBB-CSIC), 08036 Barcelona, Spain; jose.martinez@iibb.csic.es; 6 CIBER de Enfermedades Cardiovasculares (CIBERCV), Instituto de Salud Carlos III (ISCIII), 28029 Madrid, Spain; 7 Institut de Recerca Sant Pau (IR SANT PAU), 08041 Barcelona, Spain

**Keywords:** oxidative stress, NADPH oxidase 5, MMP-10, AP-1, cell migration, endothelial cells

## Abstract

NADPH oxidases (NOXs) have been described as critical players in vascular remodeling, a mechanism modulated by matrix metalloproteinases. In this study, we describe for the first time the upregulation of MMP-10 through the activation of NOX5 in endothelial cells. In a chronic NOX5 overexpression model in human endothelial cells, MMP-10 production was measured at different levels: extracellular secretion, gene expression (mRNA and protein levels), and promoter activity. Effects on cell migration were quantified using wound healing assays. NOX5 overexpression increased MMP-10 production, favoring cell migration. In fact, NOX5 and MMP-10 silencing prevented this promigratory effect. We showed that NOX5-mediated MMP-10 upregulation involves the redox-sensitive JNK/AP-1 signaling pathway. All these NOX5-dependent effects were enhanced by angiotensin II (Ang II). Interestingly, MMP-10 protein levels were found to be increased in the hearts of NOX5-expressing mice. In conclusion, we described that NOX5-generated ROS may modulate the MMP-10 expression in endothelial cells, which leads to endothelial cell migration and may play a key role in vascular remodeling.

## 1. Introduction

Oxidative stress is one of the molecular mechanisms that triggers inflammation and causes endothelial dysfunction. Among all the reactive oxygen species (ROS) sources, NADPH oxidases (NOXs) play a relevant role in the human vascular wall [1,2]. NOXs have also been proposed as key players in endothelial inflammation, with different roles depending on the specific isoform [3]. Indeed, while NOX1 and NOX2 play pathological roles in atherosclerosis [4,5,6,7,8], NOX4 seems to be protective [9,10].

NOX5 is the most recently discovered and least studied member of the NOX family. However, its regulation by vascular and inflammatory stimuli such as glucose, angiotensin II (Ang II), or interferon-γ suggests a potential role in endothelial dysfunction [11]. The NOX5 protein has been localized in immune cell-infiltrated areas of human atherosclerotic plaques [12], and both NOX5 expression and activity have been found to be increased in carotid artery disease [13]. Our group has recently linked NOX5 overexpression with endothelial dysfunction. In endothelial cells, NOX5 promotes apoptosis, mitochondrial dysfunction, and cell migration but inhibits cell proliferation [14]. We have demonstrated that NOX5 promotes an inflammatory response in immortalized human aortic endothelial cells by increasing cyclooxygenase-2 expression and prostaglandin E_2_ secretion [15].

Matrix metalloproteinases (MMPs) regulate the composition of the extracellular matrix (ECM). The balance between MMPs and their inhibitors (TIMPs) modulates vascular ECM; however, the disturbance of this balance can lead to vessel wall damage and atherosclerosis [16,17]. For instance, MMP-10 has been linked to valve calcifications in patients with aortic stenosis [18]. MMP-10 also seems to be involved in the genesis of atherosclerotic plaques. In a knock-out model for ApoE and MMP-10, a reduction in the atherosclerotic lesion size, plaque calcification, and the expression of inflammatory markers were observed. In the same study, MMP-10 expression was associated with calcified areas of atherosclerotic plaques. Additionally, MMP-10 serum levels correlated with coronary calcification in subjects with subclinical atherosclerosis [19].

ROS production derived from NOXs has been associated with MMP activity in the ECM and the vascular context. NOXs play a role in vascular structure by modulating the MMP-12/TIMP-1 expression ratio [20]. Additionally, NOX2 inhibition reduced atherosclerotic plaque development, which was accompanied by a decrease in MMP-9 activity [21]. There is scarce information on a potential relationship between NOX5 and any MMP. Nonetheless, high glucose-induced oxidative stress in human glomerular mesangial cells via NOX5 activation promoted the accumulation of ECM-related proteins (collagen I, collagen IV, and fibronectin), effects that were prevented by NOX5 silencing [22]. NOX5 expression in vascular smooth muscle cells (VSMCs) and mesangial cells had similar effects on ECM-related protein levels in diabetic Akita mice [23]. Furthermore, ECM proteins accumulated in a diabetic nephropathy model of mice expressing NOX5 in the mesangial cells [24]. Collectively, these studies indicate that NOX5 plays a role in ECM remodeling.

In a recent study, a transcriptomic array analysis showed that MMP-10 was the most upregulated MMP transcript in human aortic endothelial cells overexpressing NOX5 [25]. These data suggest that NOX5 could impact ECM remodeling by activating MMP-10 production. Interestingly, several studies demonstrate that Ang II is a key factor in ECM remodeling mediated by MMP activation [26]. Based on this evidence, we hypothesize that NOX5-derived ROS might regulate MMP-10 production, potentially modulating ECM composition.

## 2. Materials and Methods

### 2.1. Cell Culture

Human aortic endothelial cells immortalized via hTERT expression (TeloHAEC) were purchased from ATCC^®^ (American Type Culture Collection, Manassas, VA, USA). TeloHAEC were maintained and grown using vascular cell basal medium with Endothelial Cell Growth kit-VEGF (ATCC^®^) at 37 °C and 5% CO_2_. The cell medium was supplemented with penicillin, streptomycin, and gentamicin (Sigma Aldrich, Saint Louis, MO, USA). From this clonal cell line three different in vitro approaches were performed: (i) teloHAEC silenced with siRNA against NOX5 (siNOX5) or MMP-10 (siMMP-10); (ii) teloHAEC infected with NOX5-β adenoviral particles to generate an acute overexpression model; and (iii) teloHAEC transfected with a NOX5-β expression plasmid to generate a chronic overexpression model.

### 2.2. MMP-10 and NOX5 Specific Silencing

Three different siRNAs were used: siMMP-10 (sc-41555, Santa Cruz Biotechnology, Dallas, TX, USA), siNOX5 (5′-GGAGUGUGACAAUGAGAAAUC-3′) (designed by the group), and the non-targeting siCtrl (Thermo Fisher Scientific Inc.^®^). Transfection was performed using lipofectamine 3000 (Thermo Fisher Scientific Inc.^®^) and 50 nM of siRNA as a final concentration in the cell medium.

### 2.3. NOX5 Acute Overexpression Model

A NOX5-encoding adenovirus was used to generate the acute overexpression model. This adenovirus codifies for NOX5-β cDNA sequence and has previously been used in this cell line [15]. Briefly, adenoviral particles were diluted in vascular cell basal medium (ATCC^®^) supplemented with 2% fetal bovine serum and antibiotics. This solution was added to teloHAEC for 3 h at a multiplicity of infection 50 (MOI50). After that time, the solution was replaced with a fresh medium. Infection timings are calculated from the moment when adenoviruses are added to the cell culture in a solution of vascular cell basal medium.

### 2.4. NOX5 Chronic Overexpression Model

TeloHAEC were transfected with pcDNA3.2-NOX5-β expression vector [27], or with the pcDNA3.2 control vector (Mock), an approach that allowed us to generate the tHNOX5 and tHMock cell lines, respectively. Half a million teloHAEC cells were seeded in two different wells and were transfected with 1 μg of plasmid using the Lipofectamine 3000^TM^ system (L3000001, Thermo Fisher Scientific Inc.^®^, Waltham, MA, USA). Transfected cells were selected with 100 μg/mL geneticin (11811023, Gibco^TM^, Thermo Fisher Scientific Inc.^®^) for two months. Sequences of the plasmids used for cell line generation are included in the Supplementary Materials section.

### 2.5. Cell Culture Reagents

Ang II (A-9525, Sigma Aldrich) was used at a final concentration of 0.1 μM to stimulate NOX5-infected cells and 0.25 μM to stimulate the stable cell lines. The p38 MAPK inhibitor (ab145872, Abcam^®^, Waltham, MA, USA), JNK MAPK inhibitor (JNK-IN-8, SML1246, Sigma Aldrich), and ERK MAPK inhibitor (PD98059, 9900S, Cell Signaling Technology, Danvers, MA, USA) were used at final concentrations of 5 μM in the cell medium.

### 2.6. Wound Healing Assay

In this assay 500,000 cells/well were seeded overnight. The day after, three scratches were performed in each well, and pictures were taken at different timings using a Nikon SMZ18 light microscope (Nikon, Tokyo, Japan). In the cases where Ang II stimulation or MAPK inhibition was used, they were added right after making the wound. Migration capacity was measured as the % of scratched surface healed between the captured images, which was quantified using Image J^®^ (v 1.53) software (NIH, Bethesda, MD, USA).

### 2.7. RNA Isolation, Reverse-Transcription and qPCR

Total RNA was extracted from 500,000 cells seeded in 6-well plates. The cell medium was removed, and the cells were washed with pre-cold PBS (10010023, Gibco^TM^). Subsequently, 1 mL of TRIzol (15595026, Thermo Scientific^®^) was added per well. RNA was extracted using standard protocols with organic solvents and was resuspended in 20 μL of DEPC-treated water (AM9915G, Thermo Scientific^®^). One μg of each RNA sample was used for reverse transcription with SuperScript III Synthesis Kit (18080085, Thermo Scientific^®^). These cDNA samples were used for quantitative PCR (qPCR) with iQ SYBR Green (1708880, Bio-Rad^®^, Hercules, CA, USA) in an iQ5 Multicolor Real-Time PCR Detection System (Bio-Rad^®^). Glyceraldehyde 3-phosphate dehydrogenase (GAPDH) was used as a housekeeping gene, and each reaction was performed in triplicate. The specific primers for cDNA amplification are summarized in Table 1.

### 2.8. gDNA Extraction and Cell Line Genotyping

For genomic DNA (gDNA) extraction, 500,000 cells were seeded in 6-well plates overnight. The following day, trypsin was added, and cells were detached and centrifuged for 5 min at 130× *g*. Cell pellets were resuspended in 1× KAPPA Extraction Buffer, and the KAPPA^®^ Express Extract kit (KK7103, Kapa Biosystems, Merck KGaA^®^, Darmstadt, Germany) was used for gDNA obtention. DNA concentration and quality were measured using a Nanodrop ND-1000 spectrophotometer (Thermo Scientific^®^).

Conventional PCR using HotStartTaq DNA Polymerase (203205, QIAGEN^®^ GmbH, Hilden, Germany) was performed to detect if the pcDNA3.2-NOX5-β and the pcDNA3.2 control plasmids were present in the genome of these cells. Three specific primers were designed with this purpose: P1 (5′-CGTGTACGGTGGGAGGTCTA-3′), P2 (5′-CCATCTTCTCCTGCAATGGT-3′), and P3 (5′-AGGGAAGAAAGCGAAAGGAG-3′). The PCR reaction protocol consisted of the enzyme activation for 15 min at 95 °C, followed by 35 amplification cycles (20 s at 95 °C, 20 s at 60 °C, and 20 s at 72 °C). P1 is a sense primer that hybridizes with both plasmids, P2 is an antisense primer that hybridizes in the expression cassette of the pcDNA3.2-NOX5-β plasmid, and P3 is an antisense primer located after the expression cassette, common to both plasmids. This primer combination amplifies a 218 bp fragment of the pcDNA3.2-NOX5-β plasmid using P1 and P2 primers; a 449 bp fragment of the pcDNA3.2-Mock plasmid using P1 and P3 primers; and a 2818 bp fragment of the pcDNA3.2-NOX5-β plasmid using P1 and P3 primers, the latter prevented by reducing the extension time in each PCR cycle.

### 2.9. Protein Extraction and Immunodetection

For protein extraction, cells were washed with pre-cold PBS (10010023, Gibco^TM^). After that, cells were lysed by scraping in 100 μL of RIPA Buffer (1% NP-40, 150 mM NaCl, 50 mM Tris pH = 8, 0.1% SDS, and 0.5% sodium deoxycholate) supplemented with protease inhibitors (11697498001, Merck KGaA^®^). Finally, samples were sonicated, and their concentration was quantified using a Pierce^TM^ BCA Protein Assay Kit (23225, ThermoFisher^®^).

Thirty μg of proteins from each sample were diluted in RIPA buffer, and 10% β-mercaptoethanol-Laemmli buffer (1610747, BioRad^®^). Electrophoresis was performed in 10% acrylamide gels for 100 min at a constant 120 V. Then, proteins were transferred to 0.45 μm pore nitrocellulose membranes (GE10600003, Merck KGaA^®^) for 70 min at 350 mA and 4 °C. Membranes were blocked with 1% BSA (A9418, Merck KGaA^®^) in 0.05% Tween-PBS for 1 h at room temperature. The blocking solution was replaced with either 1:500 MMP-10 (MAB910, R&D Systems, Minneapolis, MN, USA) or NOX5 (191010, Abcam^®^) primary antibodies, and membranes were incubated at 4 °C overnight. Membranes were washed three times with 0.05% Tween-PBS solution and incubated for 60 min with the pertinent secondary antibody diluted 1:2000 in blocking solution. Secondary antibodies against rabbit (NA934V, GE Healthcare, Merck KGaA^®^) and mouse (NA931V, GE Healthcare, Merck KGaA^®^) Ig constant fractions were used. Finally, membranes were washed three times, and blots were visualized with ECL Prime Western Blotting Detection Reagent^TM^ (GE28980926, Merck KGaA^®^). β-actin protein was used for protein expression normalization, with the primary antibody (A5441, Sigma Aldrich^®^) diluted 1:10,000 in 5% non-fat milk in 0.05% Tween-PBS solution. Blots were quantified using Image J^®^ software (v1.53, NIH, Bethesda, MD, USA). Protein expression levels were expressed as the protein ratio over β-actin of each sample. In the case of the in vivo samples, heart lysates were incubated with the MMP-10 primary antibody (ab261733, Abcam^®^) at 1:500 in 5%BSA in 0.05% Tween-PBS solution.

### 2.10. Extracellular MMP-10 Detection by ELISA

Supernatant samples from cell cultures were obtained from wells containing 500,000 teloHAEC in 1.5 mL of complete medium. In order to produce a reliable measurement over time, 50 μL of supernatant was removed from each well after 6, 12, 24, 36, and 48 h. These samples were diluted 1:4 in PBS (10010023, Gibco^TM^) before being assayed using the Human Total MMP-10 DuoSet ELISA kit (DY910, R&D Systems^®^).

### 2.11. MMP-10 Promoter Activity Assays

Previously, a 2.0-kb fragment of the human MMP-10 promoter in a pGL-3 vector expressing the Firefly luciferase was cloned and several constructions were generated [28]. This promoter was transfected together with a Renilla luciferase reporter plasmid in tHMock and tHNOX5 cell lines. Luciferase activity was measured in cell lysates using a tube luminometer and the Luciferase Assay Kit (E1501, Promega^®^, Madison, WI, USA) following the manufacturer’s indications. Results were calculated as the ratio between Firefly and Renilla luciferase activities.

### 2.12. ROS Production

ROS production was measured using the AmpliFluRed^TM^ kit (90101, Sigma Aldrich^®^) in intact cells cultured in 96-well format plates. The different stimuli were added in 50 μL of Krebs–Ringer buffer (K4002, Merck KGaA^®^) for 5 min. These stimuli were ionomycin (Io) (I0634, Sigma Aldrich^®^), phorbol 12-myristate 13-acetate (PMA) (P1585, Sigma Aldrich^®^), and Ang II. After the incubation time, following manufacturer instructions, fluorescence intensity was measured at 544 nm excitation and 590 nm emission wavelengths in a microplate fluorescence reader (PolarStar^®^, BMG Labtech, Ortenberg, Germany).

### 2.13. Real-Time Proliferation Measurement

Proliferation was measured using the Real-Time Cell Analyzer xCELLigence (Agilent Technologies, Santa Clara, CA, USA). Briefly, 15,000 endothelial cells were seeded per well in the assay plates provided by the manufacturer. The automatic monitoring was performed every 15 min for a total time of 48 h.

### 2.14. In Vivo Studies

In vivo experiments were performed in accordance with European Community Council Directives (2010/63/EU) guidelines for the care and use of laboratory animals and were approved by the University of Navarra Animal Research Review Committee (Protocol 106-17). Mice were maintained in the conventional animal facility of the Universidad de Navarra, with controlled temperature and humidity conditions, fed ad libitum, and with 12-h light cycles. Our in vivo model consisted of a conditional endothelial knock-in model in a C57BL6/J male. As NOX5 is absent in the rodent genome, this model introduced in an inducible manner the human NOX5 protein in endothelial cells. A transgenesis process, and thus the expression of NOX5 protein in endothelial cells was induced with three tamoxifen injections in 3 doses at non-consecutive days at a dose of 40 mg/kg/day.

### 2.15. Statistical Analysis

Firstly, data normality was studied to check if the results followed a parametric distribution. Then, in the case of the 2 group comparisons, a *t*-test or Mann–Whitney U analysis was used. However, in the case of 4 or more group comparisons generated from the analysis of 2 variables, two-way ANOVA tests were used, and the effect of each variable, the interaction between them, and group differences were analyzed. In the specific case of time-dependent MMP-10 ELISAs, a paired *t*-test was used, and the area under the curve was also analyzed. Results were expressed as median and interquartile range (IQR) or as mean ± standard error of the mean (SEM), and statistical significance was established as *p* < 0.05. For statistical analysis and graphical illustrations of the results, GraphPad Prism 8 was used (GraphPad^®^, San Diego, CA, USA).

## 3. Results

### 3.1. NOX5 Enhances MMP-10 Production and Cell Migration in Human Endothelial Cells

**Figure 1:**
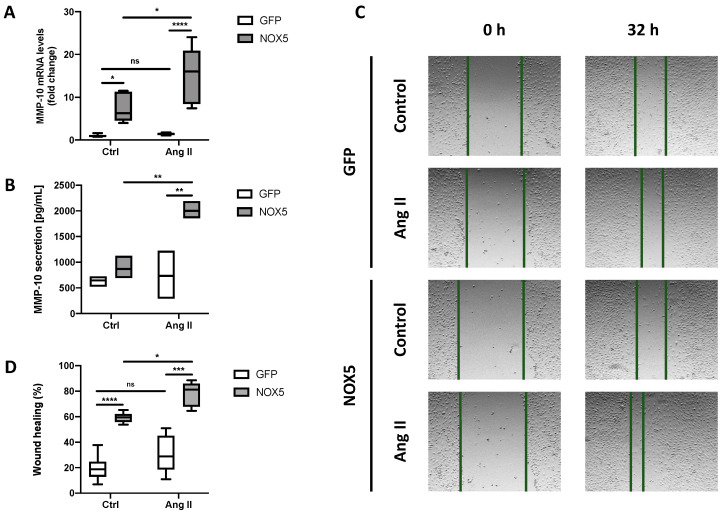
**NOX5-β promotes MMP-10 production and cell migration in endothelial cells:** (**A**) MMP-10 mRNA levels after 24 h of infection and 16 h of Ang II stimulation (*n* = 6). (**B**) MMP-10 protein levels in cell supernatants after 24 h of infection and 24 h of Ang II stimulation (*n* = 3). (**C**) Representative images of the wound-healing assay of teloHAEC after 24 h of infection and Ang II stimulation. The wound was performed 24 h after the infection when Ang II was added (0 h) and was monitored for 32 h. (**D**) Wound-healing assay quantification (*n* = 6). GFP: teloHAEC infected with GFP-encoding adenovirus. NOX5: teloHAEC infected with NOX5-encoding adenovirus. Ctrl: non-stimulated cells. Ang II: cells stimulated with 0.1 μM Ang II. ns: not significant differences. * *p* < 0.05, ** *p* < 0.01, *** *p* < 0.001, **** *p* < 0.0001. Data are presented as median and IQR.

Initially, we studied whether NOX5 overexpression could promote MMP-10 upregulation in endothelial cells. TeloHAEC infection with NOX5 adenovirus produced a five-fold increase in MMP-10 mRNA levels, an effect that increased up to 15-fold when Ang II was added (Figure 1A). The extracellular secretion of MMP-10 protein also increased in cells infected with NOX5 adenovirus, although this increase was only statistically significant in cells stimulated with Ang II (Figure 1B). Cell migration was studied as a phenotype modulated by MMPs-related genes. As shown in Figure 1C,D, NOX5 accelerated teloHAEC migration, which was even faster when Ang II was added. Finally, other MMP-related genes such as MMP-14, TIMP-1, and TIMP-2 were upregulated through Ang II activation in NOX5-infected cells (Figure S1). These results suggest that, besides MMP-10, NOX5 can regulate the expression of other MMPs.

### 3.2. MMP-10 Is Involved in Cell Migration in Human Endothelial Cells

**Figure 2:**
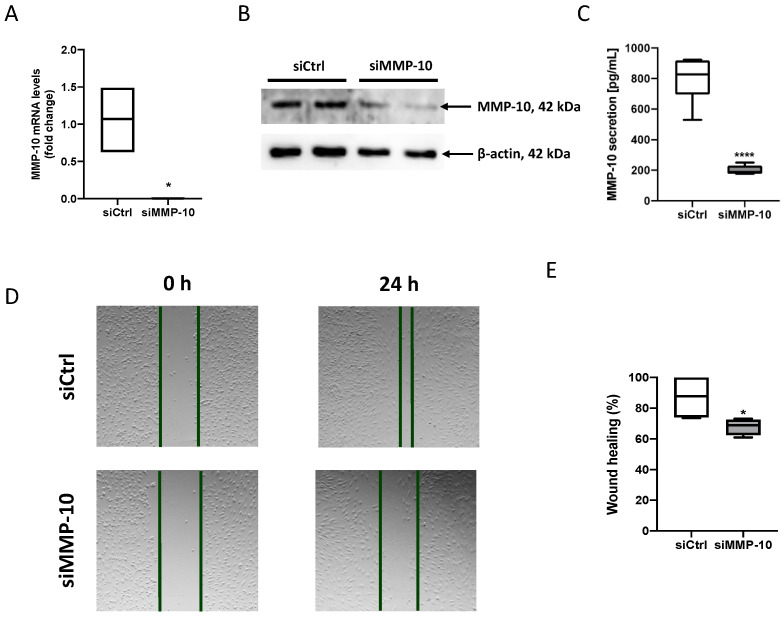
**MMP-10 silencing decreases MMP-10 expression and secretion as well as endothelial cell migration:** (**A**) MMP-10 mRNA levels 24 h after MMP-10 silencing (*n* = 3). (**B**) Immunoblot of MMP-10 and β-actin 24 h after silencing (*n* = 2). (**C**) MMP-10 protein levels in cell supernatants 24 h after silencing. (**D**) Representative images of teloHAEC silenced with siCtrl or siMMP-10 0 h and 48 h after the scratch of the wound-healing assay. (**E**) Quantification of the wound-healing assay (*n* = 6). siCtrl: teloHAEC silenced with a Ctrl siRNA. siMMP-10: teloHAEC silenced with a siMMP-10. * *p* < 0.05, **** *p* < 0.0001. Data are presented as median and IQR.

To further investigate whether MMP-10 could be involved in endothelial cell migration, MMP-10 expression was silenced in teloHAEC using a specific siRNA (siMMP-10). siMMP-10 effectively reduced MMP-10 expression at mRNA, as well as intracellular and secreted protein levels in teloHAEC (Figure 2A–C). Furthermore, MMP-10 silencing reduced teloHAEC migratory capacity compared with cells transfected with a control siRNA (siCtrl) (Figure 2D,E). These results indicate that MMP-10 is involved in TeloHAEC migration.

### 3.3. NOX5 Chronic Overexpression Enhances MMP-10 Production and the Associated Cell Migration in Human Endothelial Cells

The NOX5 adenoviral model in TeloHAEC could be considered extreme, far from the pathophysiological range of NOX5 overexpression that could be found in human health and disease [15]. For that reason, to better mimic the pathophysiological levels of this oxidase, an alternative chronic overexpression model was developed. We generated a chronic NOX5 overexpression cell line (tHNOX5) and the respective control line (tHMock). These cells were selected and grown after their transfection with the pertinent plasmids until stably expressing cell lines were obtained. First, we demonstrated that the plasmids were integrated into the gDNA of tHMock and tHNOX5 cells using PCR (Figure S2A). Second, a significant increase in NOX5 (both mRNA and protein) was found in tHNOX5 cells (Figure S2B–D). Third, ROS production was higher in tHNOX5 than in tHMock cells, although this increase was not statistically significant. Nevertheless, ROS production increased in response to different pharmacological stimuli such as Io or PMA in tHNOX5 cells (Figure S2E–G). Finally, several pro-oxidant and antioxidant enzymes were analyzed to assess NOX5 effects on redox homeostasis. Within the NOX family, NOX5 overexpression produced an increase in NOX4 mRNA expression (Figure S3A–C). Within the antioxidant enzymes, NOX5 overexpression produced an increase in SOD2 expression (Figure S3E). Collectively, these data demonstrate that the tHNOX5 cell line stably overexpresses a functional NOX5 protein that responds to pharmacological stimuli (PMA and Io) by increasing ROS generation.

**Figure 3:**
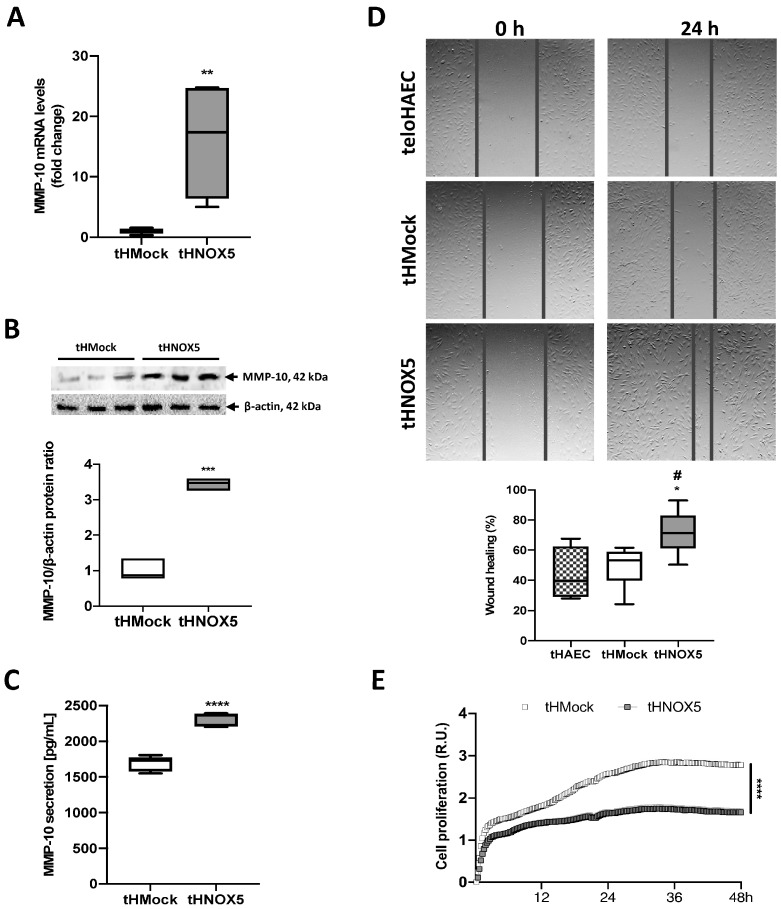
**NOX5 chronic overexpression promotes MMP-10 expression and endothelial cell migration:** (**A**) MMP-10 mRNA levels of tHMock and tHNOX5 cell lines (*n* = 6). (**B**) MMP-10 and β-actin immunoblots of tHMock and tHNOX5 cell lines and their quantification (*n* = 3). (**C**) MMP-10 protein levels in cell supernatants from tHMock and tHNOX5 cultures (*n* = 6). (**D**) Representative images of teloHAEC, tHMock, and tHNOX5 cells in the wound-healing assay 0 h and 24 h after the scratch and their quantification (*n* = 6). (**E**) Cell proliferation level of tHMock and tHNOX5 cells measured using xCelligence technology (*n* = 6). * *p* < 0.05 vs. tHMock cell line, ** *p* < 0.01 vs. tHMock cell line, *** *p* < 0.001 vs. tHMock cell line, **** *p* < 0.0001 vs. tHMock cell line, ^#^ *p* < 0.05 vs. teloHAEC cell line. Data are presented as median and IQR.

Next, we found that tHNOX5 cells exhibited increased MMP-10 expression (Figure 3A,B). This upregulation was accompanied by enhanced MMP-10 secretion to the extracellular medium (Figure 3C). Finally, the tHNOX5 cell line presented an increase in endothelial cell migration in the wound-healing assay (Figure 3D). Interestingly, tHNOX5 cells presented significantly lower proliferation levels compared to tHMock cells (Figure 3E), supporting the idea of a promigratory phenotype of these cells.

Further, we found that the tHNOX5 cell line expressed higher levels of MMP-1 and MMP-2 and lower levels of TIMP-1 and TIMP-2 compared to the tHMock control line (Figure S3F–H). These data demonstrate that chronic NOX5 overexpression increases MMP-10 production and modulates other MMP-related genes, which could impact endothelial cell migration.

**Figure 4:**
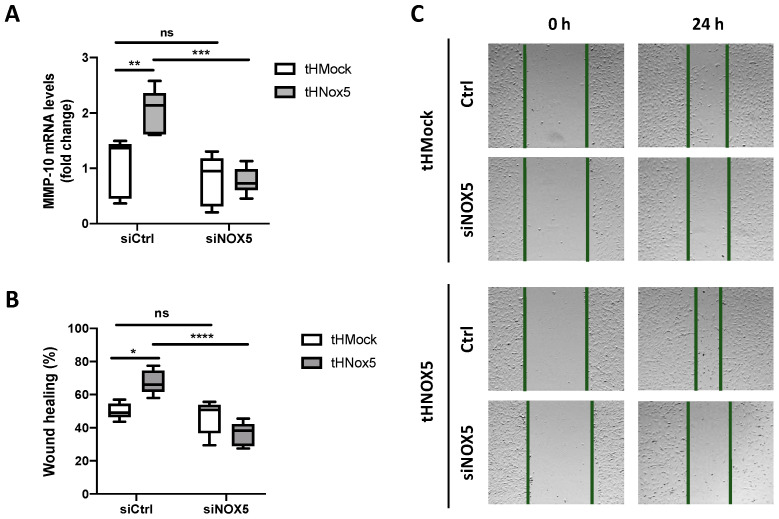
**NOX5 silencing in the NOX5 chronic overexpression model attenuates MMP-10 expression and endothelial cell migration:** (**A**) MMP-10 mRNA levels of tHMock and tHNOX5 cells transfected with a control siRNA (siCtrl) or a siRNA against NOX5 (siNOX5) (*n* = 6). (**B**,**C**) Quantification of the wound-healing assay (*n* = 6) (**B**) and representative images 0 h and 24 h after the scratch (*n* = 6) of tHMock and tHNOX5 cells transfected with siCtrl or siNOX5. ns: not significant differences, * *p* < 0.05, ** *p* < 0.01, *** *p* < 0.001, **** *p* < 0.0001. Data are presented as median and IQR.

To verify whether NOX5 chronic overexpression is involved in MMP-10 upregulation and cell migration, NOX5 was silenced in tHMock and tHNOX5 cells using a specific siRNA (siNOX5). NOX5 silencing reduced MMP-10 expression in tHNOX5 cells, reaching levels similar to those of tHMock cells (Figure 4A). Interestingly, silencing abrogated the increased migratory capacity exhibited by tHNOX5 cells, supporting a key role of NOX5 in this phenotype (Figure 4B,C).

**Figure 5:**
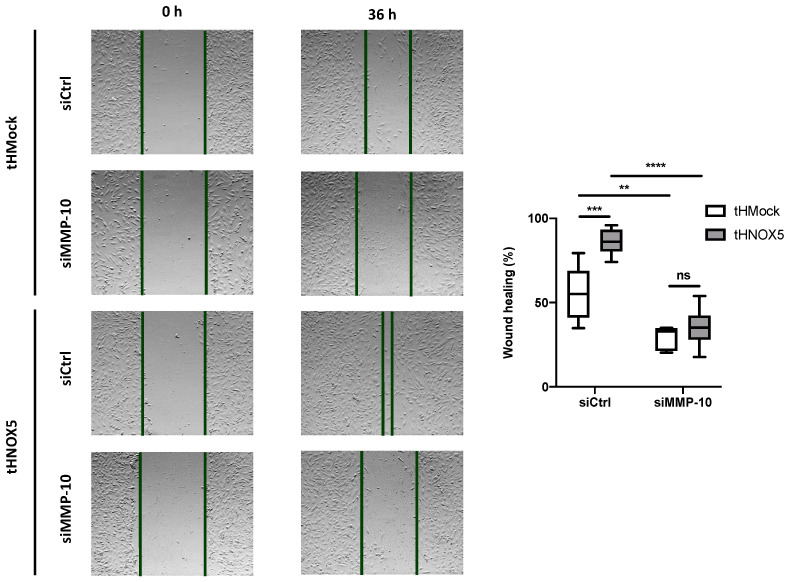
**MMP-10 silencing in the NOX5 chronic overexpression model attenuates enhanced endothelial cell migration.** (**Left panel**): Representative images of tHMock and tHNOX5 cells silenced with siMMP-10 0 h and 36 h after the scratch of the wound-healing assay. (**Right panel**): Quantification of the wound-healing assay of tHMock and tHNOX5 cell lines silenced with siMMP-10 (*n* = 6). tHMock: stable cell line transfected with pcDNA3.2-Mock. tHNOX5: stable cell line transfected with pcDNA3.2-NOX5. siCtrl: teloHAEC silenced with a Ctrl siRNA. siMMP-10: teloHAEC silenced with a siMMP-10. ns: not significant differences, ** *p* < 0.01, *** *p* < 0.001, **** *p* < 0.0001. Data are presented as median and IQR.

siMMP-10 was also used to study whether MMP-10 expression plays a role in the enhanced promigratory activity exhibited by tHNOX5 cells. tHNOX5 cells silenced against MMP-10 expression lost their increased ability to migrate (Figure 5). All these data strongly suggest that NOX5 chronic overexpression promotes cell migration via MMP-10 upregulation in human endothelial cells.

### 3.4. Ang II Stimulation of NOX5-Overexpressing Human Endothelial Cells Enhances MMP-10 Production and Cell Migration

**Figure 6:**
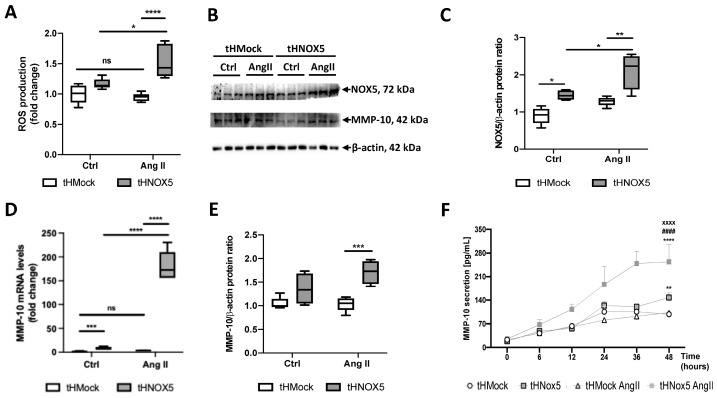
**Ang II potentiates MMP-10 upregulation promoted by the chronic overexpression of NOX5:** (**A**) Effect of Ang II stimulation on the production of ROS by tHMock and tHNOX5 cells (*n* = 6). (**B**) Representative image of NOX5, MMP-10, and β-actin immunoblots of tHMock and tHNOX5 cell lines after 16 h of Ang II stimulation. (**C**) Quantitative analysis of NOX5 protein expression of tHMock and tHNOX5 cell lines after 16 h of Ang II stimulation (*n* = 6). (**D**) MMP-10 mRNA levels of tHMock and tHNOX5 cell lines after 8 h of Ang II stimulation (*n* = 6). (**E**) Quantitative analysis of MMP-10 protein expression of tHMock and tHNOX5 cell lines after 16 h of Ang II stimulation (*n* = 6). (**F**) MMP-10 protein levels in tHMock and tHNOX5 cultures at different times after Ang II stimulation (*n* = 6). tHMock: stable cell line transfected with pcDNA3.2-Mock. tHNOX5: stable cell line transfected with pcDNA3.2-NOX5. ns: not significant differences, * *p* < 0.05, ** *p* < 0.01, *** *p* < 0.001, **** *p* < 0.0001 vs. tHMock. ^####^ *p* < 0.0001 vs. tHNOX5. ^xxxx^ *p* < 0.0001 vs. tHMock Ang II. Data are presented as median and IQR or as mean ± SEM.

Next, we evaluate the role of Ang II in the chronic overexpression model. Ang II stimulation increased extracellular ROS production by 50% in tHNOX5 cells, while it had no effect under these conditions in tHMock cells (Figure 6A). Accordingly, Ang II stimulation increased protein levels of NOX5 in tHNOX5 cells, while it had no effect under these conditions in tHMock cells (Figure 6B,C). Then, the effect of the Ang II-derived NOX5 stimulation on MMP-10 expression was studied. A synergistic effect was found between NOX5 and Ang II in tHNOX5 cells. At baseline, MMP-10 mRNA levels were higher in tHNOX5 cells compared to tHMock cells; however, they strongly increased in response to Ang II (Figure 6B,D). Finally, although Ang II only had a minor effect on MMP-10 protein levels (Figure 6E), it significantly potentiated the increase in MMP-10 secretion promoted by NOX5 (Figure 6F). Interestingly, overexpression of NOX5 in tHNOX5 cells induced upregulation of Ang II type 1 (AT1R) and type 2 (AT2R) receptors (Figure S4), suggesting that overexpression of NOX5 would increase the sensitivity of these cells to Ang II stimulation.

Later, tHMock and tHNOX5 cells were stimulated with Ang II to study its effect on cell migration. Ang II promoted endothelial cell migration and exerted a synergistic effect with NOX5 overexpression (Figure S5). This result indicates that Ang II promotes endothelial cell migration in NOX5-overexpressing cells.

### 3.5. ERK, p38, and JNK Are Involved in the Ang II/NOX5/MMP-10/Cell Migration Pathway

In previous studies, our group showed that MAPKs play a key role in MMP-10 upregulation in human aorta endothelial cells (HAECs) stimulated with thrombin [28]. Therefore, we tested whether MAPKs are involved in the Ang II/NOX5/MMP-10/cell migration axis. Firstly, the migratory capacity of tHMock and tHNOX5 cells was studied in the presence of 219138-24-6 (p38 MAPK inhibitor), PD98059 (MEK/ERK inhibitor), or JNK-IN-8 (JNK inhibitor). Interestingly, the promigratory phenotype of the tHNOX5 cell line was prevented with each one of the inhibitors (Figure S6). Next, we studied the effect of these inhibitors on MMP-10 mRNA levels in these cells.

**Figure 7:**
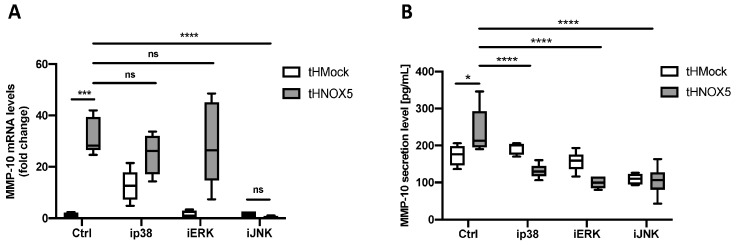
**p38 MAPK, MEK/ERK, and JNK pathways participate at different levels in NOX5-dependent regulation of MMP-10:** (**A**) MMP-10 mRNA levels of tHMock and tHNOX5 cell lines after 24 h of incubation with 5 μM 219138-24-6 (p38 MAPK inhibitor, ip38), PD98059 (MEK/ERK inhibitor, iERK), or JNK-IN-8 (JNK inhibitor, iJNK) (*n* = 6). (**B**) MMP-10 protein in the conditioned medium of tHMock and tHNOX5 cell lines after 24 h of incubation with 5 μM 219138-24-6 (ip38), PD98059 (iERK), or JNK-IN-8 (iJNK) (*n* = 6). tHMock: stable cell line transfected with pcDNA3.2-Mock. tHNOX5: stable cell line transfected with pcDNA3.2-NOX5. n.s.: not significant differences, * *p* < 0.05, *** *p* < 0.001, **** *p* < 0.0001. Data are presented as median and IQR.

The inhibition of p38 MAPK or MEK/ERK pathways had no effect on MMP-10 mRNA levels (Figure 7A). However, in tHNOX5 cells, JNK-IN-8 reduced MMP-10 expression to levels similar to those detected in tHMock cells (Figure 7A). These results demonstrate that the JNK pathway is involved in MMP-10 upregulation by NOX5 overexpression. All three inhibitors, however, were able to prevent the increased secretion of MMP-10 by tHNOX5 cells (Figure 7B). These data suggest that p38 MAPK and MEK/ERK may be involved in MMP-10 activation and/or extracellular secretion. A similar result was obtained in cells stimulated with Ang II (Figure S7).

### 3.6. NOX5 Enhances MMP-10 Promoter Activity via JNK and AP-1

Finally, to study the regulation of MMP-10 at the transcriptional level, different luciferase reporter expression plasmids under the control of human MMP-10 promoters were used in transient transfection assays [28].

**Figure 8:**
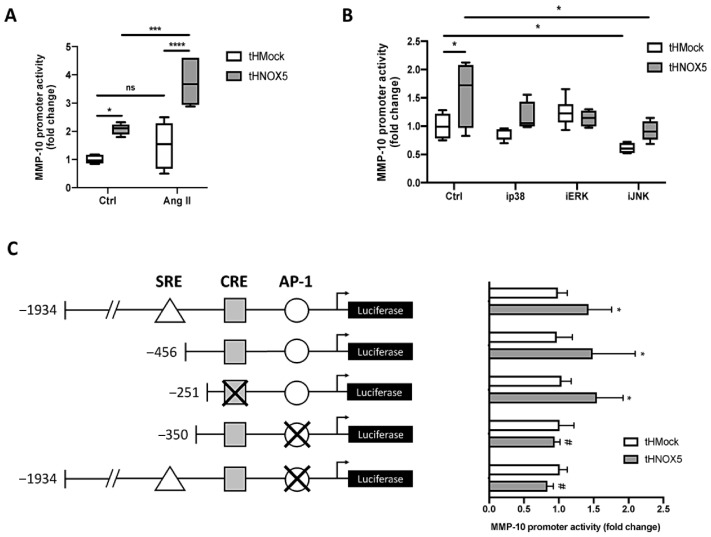
**NOX5 enhances MMP-10 promoter activity via the JNK pathway and a functional AP-1 site:** (**A**) MMP-10 promoter activity of tHMock and tHNOX5 cell lines at baseline and stimulated with 0.25 μM Ang II (*n* = 6). (**B**) MMP-10 promoter activity of tHMock and tHNOX5 cell lines after 24 h of incubation with 5 μM 219138-24-6 (p38 MAPK inhibitor, ip38), PD98059 (MEK/ERK inhibitor, iERK), or JNK-IN-8 (JNK inhibitor, iJNK) (*n* = 6). (**C**) Schematic representation of MMP-10 promoter constructions used and quantification of MMP-10 promoter activity of tHMock and tHNOX5 cell lines transfected with MMP-10 promoter constructions (*n* = 6). CREB: cAMP response element binding protein (CREB) putative union site. AP-1: activator protein-1 (AP-1) putative union site. Crosses indicate site-directed mutations in the putative union sites. tHMock: stable cell line transfected with pcDNA3.2-Mock. tHNOX5: stable cell line transfected with pcDNA3.2-NOX5. ns: not significant differences, * *p* < 0.05 vs. tHMock Ctrl, *** *p* < 0.001, **** *p* < 0.0001, ^#^ *p* < 0.05 vs. tHNOX5 Ctrl cells. Data are presented as median and IQR or as mean ± SEM.

Firstly, tHNOX5 cells exhibited higher MMP-10 promoter activity than tHMock cells, and Ang II further increased it (Figure 8A). Secondly, JNK-IN-8, but not 219138-24-6 or PD98059, reduced the activity of MMP-10 promoter in both tHMock and tHNOX5 cells (Figure 8B). Thirdly, cells were transfected with a combination of MMP-10 promoter constructs with mutations and deletions of different putative binding regions for AP-1 (activator protein 1, Jun/Fos heterodimer) and CREB (cAMP response element binding protein) transcription factors (Figure 8C). The mutation of the CRE site did not affect the increased MMP-10 promoter activity detected in tHNOX5 cells. However, when the AP-1 site was mutated, the activity levels of the MMP-10 promoter of tHNOX5 cells were reduced to those present in tHMock cells.

These results indicate that NOX5 upregulates MMP-10 expression through a transcriptional mechanism involving a functional AP-1 site.

### 3.7. Endothelial NOX5 Expression Leads to Cardiac MMP-10 Upregulation at Protein Levels

**Figure 9:**
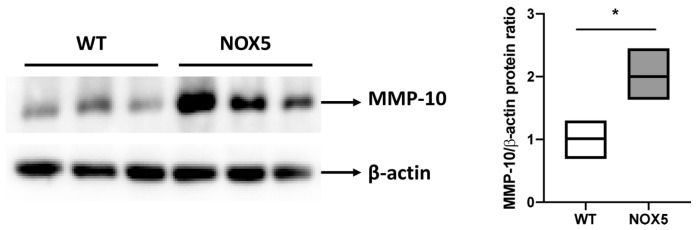
**Endothelial NOX5 expression increases cardiac MMP-10 protein levels in mice.** (**Left**): Representative immunoblot of MMP-10 and β-actin proteins in hearts of WT and NOX5 mice. (**Right**): Quantification of cardiac MMP-10 protein levels in WT and NOX5 mice (*n* = 3). WT: wild-type mice. NOX5: endothelial NOX5-expressing mice. * *p* < 0.05 vs. WT mice. Data are presented as median and IQR.

Finally, MMP-10 levels were studied in our endothelial NOX5 knock-in murine model. At the cardiac level, NOX5-expressing mice presented an increase in MMP-10 protein expression compared to wild-type mice (Figure 9).

## 4. Discussion

The key results that arise from this work may be summarized as follows: (i) NOX5 overexpression increases MMP-10 expression and extracellular secretion in teloHAEC; (ii) the NOX5-MMP-10 axis promotes teloHAEC migration; (iii) JNK and AP-1 are the regulatory factors involved in this axis; and (iv) Ang II enhances the effects of NOX5 overexpression on MMP-10 expression and extracellular secretion and migration in teloHAEC. For the first time, cross-talk between NOX5 and the MMP-10-modulating cell phenotype is described in the vascular context.

Vascular remodeling is a pathophysiological characteristic of cardiovascular diseases, which manifests itself in association with alterations of the extracellular matrix and the proteins that regulate it, the MMPs. Recently, our group and others have confirmed the relevant role of MMP-10 in the pathophysiology of atherosclerosis. For example, MMP-10 deletion in an atherosclerotic model reduced plaque progression. Moreover, in human plasma, MMP-10 levels correlate with plaque calcification in patients with subclinical atherosclerosis [20]. Likewise, plasma levels of MMP-10 correlate with neurological damage and mortality in patients with cerebrovascular disease [29] and with aortic valve calcification [19]. Among inflammatory vascular stimuli, C-reactive protein [17] and thrombin [28] upregulate MMP-10 expression, thus being able to promote the pathophysiological process of atherothrombotic disease. Inflammatory stimuli promote their effects, among other mechanisms, favoring the accumulation of ROS that alter redox signaling in cells and promote oxidative stress [30]. In this sense, we have previously reported that overexpression of NOX5 in HAEC cells upregulated several MMPs, especially MMP-10 [25]. The results of our present study confirm that NOX5 increases MMP-10 expression and secretion in endothelial cells, which are involved in endothelial cell migration. These data indicate that NOX5 may contribute to changes in the vascular ECM. In accordance with this, Casas et al. have shown that NOX5 limits post-reperfusion benefits in stroke by promoting blood–brain barrier breakdown [31].

Previously, NOXs have been related to MAPK in the vasculature [32,33,34], as well as other groups already described this MAPKs/NOX5 relationship in the vessel wall for VSMCs [35] and for endothelial cells [36]. In fact, the AP-1-mediated activation by NOX5 was already described in porcine cardiac smooth muscle cells [37]. Interestingly, we have previously demonstrated MMP-10 promoter activation via AP-1 in response to thrombin in endothelial cells [28]. In the present study, we extended these results demonstrating that NOX5 promotes AP-1 activation via JNK, a ROS-sensitive pathway. The activation of this JNK/AP-1 pathway may be responsible for the NOX5/MMP-10-dependent endothelial cell migration.

Ang II contributes significantly to the pathophysiology of vascular diseases, among other pathways, by modulating the activity of the NOX family [38]. Ang II has been described to promote cell migration by NOX/MMP pathways. For instance, Ang II promoted VSMC migration and proliferation by switching on the NOX1-MMP-9 pathway [39]. On the other hand, Ang II induced vascular remodeling in adventitial fibroblasts and VSMCs by promoting cell migration via NOX2 [40,41,42]. In this sense, NOX2 expressed by fibroblasts seems to regulate the hypertensive response and arterial wall resistance in vivo [43]. Likewise, Ang II is involved in the regulation of the activity of NOX5 [11,44]. For instance, Ang II induces enhanced ventricular hypertrophy, interstitial fibrosis, and contractile dysfunction in a cardiomyocyte-specific NOX5 knock-in mouse model [45]. In this context, our results showed that Ang II enhanced MMP-10 production/secretion and extracellular migration in NOX5-overexpressing endothelial cells, either acutely (TeloHAEC cells infected with NOX5 adenoviral vectors) or chronically (tHNOX5 cells). Interestingly, overexpression of NOX5 in tHNOX5 cells upregulated AT1R and AT2R, suggesting that NOX5 plays a key role in the responsiveness of these cells to Ang II by increasing their sensitivity to this hormone. In tHNOX5, Ang II could be acting mainly on the overexpressed NOX5, since the levels of endogenous NOX5 are almost null. In fact, Ang II did not exert relevant effects on ROS production, NOX5 expression, and MMP10 expression/secretion in tHMock cells. However, we cannot rule out that due to the overexpression of exogenous NOX5, other NOX family members regulated by Ang II could be upregulated [46]. In this sense, our data show that NOX5 overexpression upregulated NOX4 in tHNOX5 cells. Finally, NOX5 overexpression could positively regulate other pro-oxidant systems that respond to Ang II-mediated signaling. Regardless, we demonstrated that a characteristic stimulus of numerous cardiovascular diseases, including arterial hypertension and atherosclerosis, could promote ECM remodeling through the NOX5/MMP-10 axis.

Overexpression of Nox5 increased the migration of tHNOX5 compared to tHMock cells, closely associated with the upregulation of MMP-10. In favor of a key role of MMP-10 in this process, the silencing of MMP-10 reduced the migratory capacity of TeloHAEC cells, as well as the tHNOX5 cells, up to levels similar to those of the tHMock cells. On the other hand, Ang II increased the migration of cells in both cell lines, although this effect was higher in tHNOX5 cells. Interestingly, Ang II upregulated other MMP-related genes (MMP-1, MMP14, TIMP-1, and TIMP-2) in both tHMock and tHNOX5 cells, although this effect was again greater in tHNOX5 cells. The greater effect of Ang II on these MMP-related genes in tHNOX5 cells could be due to the action of this hormone directly on the overexpressed NOX5. We can not discard that Ang II could exert its effect on other genes activated downstream in response to NOX5 overexpression. In fact, stimulation with Ang II increased the migratory capacity of both immortalized cell lines, although the effect was more quantitative in tHOX5 than in tHMock cells.

This NOX5-derived MMP-10 secretion may have consequences in different cardiovascular diseases in vivo. We have previously reported that mice expressing endothelial NOX5 did not show any change in blood pressure compared to the control group in either young or aged animals [47]. However, in another knock-in model in which NOX5 was overexpressed in endothelial and immune cells, aged NOX5 mice (68–87 weeks) exhibited an increase in blood pressure compared to wild-type mice [48]. Interestingly, although endothelial NOX5 overexpression did not affect blood pressure in our model, NOX5 produced changes in MMP-10 protein levels in the cardiac tissue. This increase in MMP-10 may alter the ECM composition of the vessel wall. Baraibar-Churio et al. described that MMP-10 deficiency aggravated the dystrophic phenotype in transgenic mice [49]. This means that the NOX5/MMP-10 axis could have a preconditioning effect, preventing pathological alterations produced by Ang II or other stimuli. Supporting this preconditioning role, our endothelial NOX5 in vivo model was demonstrated to regulate pro-inflammatory genes in both cardiac and cerebral tissues [15,47]. New studies should be developed to deep into this role of NOX5 in vivo.

## 5. Conclusions

To sum up, we found that MMP-10 production is upregulated by NOX5-mediated ROS in endothelial cells. The NOX5-MMP-10 axis impairs endothelial cell migration, being JNK and AP-1 key regulatory factors involved in this process. Interestingly, Ang II enhances the effects of NOX5 overexpression on MMP-10 expression and migration of teloHAEC. In addition, NOX5 endothelial expression increases MMP-10 cardiac protein levels in mice. Collectively, these results allow us to speculate on a possible role of NOX5 in the composition of the vascular ECM, which could lead to the establishment of endothelial dysfunction and the development of complications in cardiovascular diseases.

## Figures and Tables

**Table 1:** Specific primers were used for qPCR amplification.

Gene Name	Accession Number		Sequence (5′-3′)	Product Size (bp)	Annealing T (°C)
*NOX1*	NM_007052.5	Forward	CTACCTCCCACCCCAAGTCT	227	60
		Reverse	TGACTGCTCAAACCTGACGA		
*NOX2*	NM_000433.4	Forward	CTGTGAATGAGGGGCTCTCC	340	60
		Reverse	GCAATGGTGTGAATCGCAGA		
*NOX4*	NM_016931.5	Forward	CTGTATTTTCTCAGGCGTGCAT	113	60
		Reverse	CCTCATCTCGGTATCTTGCTGC		
*NOX5*	NM_024505.4	Forward	TAAGAGGCTGTCGAGGAGTGT	71	60
		Reverse	CCAAAAGTATCTCAGAGCCCTTG		
*SOD1*	NM_000454.5	Forward	GAAGAGAGGCATGTTGGAGAC	240	59
		Reverse	GAATGTTTATTGGGCGATCC		
*SOD2*	NM_000636.4	Forward	GTTGGCCAAGGGAGATGTT	171	61
		Reverse	TCAAAGGAACCAAAGTCACG		
*MMP-1*	NM_002421.4	Forward	CAAAGGGAATAAGTACTGGGCTGT	375	60
		Reverse	TTCCTGCAGTTGAACCAGCT		
*MMP-2*	NM_004530.6	Forward	GCGGTCACAGCTACTTCTTC	153	59
		Reverse	TATCGAAGGCAGTGGAGAGG		
*MMP-10*	NM_002425.3	Forward	TTGCAGTTAAAGAACATGGAGACT	181	59
		Reverse	GAGTGGCCAAGTTCATGAGC		
*MMP-14*	NM_004995.4	Forward	TCCAGCAACTTTATGGGGGT	130	59
		Reverse	TTCCCGTCACAGATGTTGGG		
*TIMP-1*	NM_003254.3	Forward	AGAGACACCAGAGAACCCACC	373	61
		Reverse	GCAAGAGTCCATCCTGCAGT		
*TIMP-2*	NM_003255.5	Forward	CAGATGTAGTGATCAGGGCCAA	201	59
		Reverse	TCTTTCCTCCAACGTCCAGC		
*AT1R*	NM_000685.5	Forward	TCGGCACCAGGTGTATTT	245	60
		Reverse	GCCACAGTCTTCAGCTTCAT		
*AT2R*	NM_000686.5	Forward	GAAGAAGGCATAAGAACTAGGAGC	384	60
		Reverse	CACAGGTCCAAAGAGCCAGT		
*GAPDH*	NM_001289726.1	Forward	CCAAGGTCATCCATGACAAC	157	59
		Reverse	TGTCATACCAGGAAATGAGC		

*NOX1*: NADPH oxidase 1. *NOX2*: NADPH oxidase 2. *NOX4*: NADPH oxidase 4. *NOX5*: NADPH oxidase 5. *SOD1*: superoxide dismutase 1. *SOD2*: superoxide dismutase 2. *MMP-1*: matrix metalloproteinase 2. *MMP-2*: matrix metalloproteinase 2. *MMP-10*: matrix metalloproteinase 10. *MMP-14*: matrix metalloproteinase 14. *TIMP-1*: metalloproteinase inhibitor 1. *TIMP-2*: metalloproteinase inhibitor 2. *AT1R*: angiotensin II type 1 receptor. *AT2R*: angiotensin II type 2 receptor. *GAPDH*: Glyceraldehyde 3-phosphate dehydrogenase.

## Data Availability

Data will be made available on request.

## Supplementary Materials

The following supporting information can be downloaded at: https://www.mdpi.com/article/10.3390/antiox13101199/s1, Figure S1: Ang II-mediated NOX5-β stimulation upregulates genes related to extracellular matrix remodeling; Figure S2: The stable teloHAEC cell line expressing NOX5 (tHNOX5) serves as model of chronic NOX5 overexpression; Figure S3: NOX5 chronic overexpression affects other redox and MMP-related genes; Figure S4: NOX5 chronic overexpression seems to affect Ang II receptors (AT1R and AT2R) expression, with a more intense effect on ATR2; Figure S5: Ang II potentiates the endothelial cell migration of NOX5-overexpressing cells. NOX5; Figure S6: MAPK, MEK/ERK, and JNK pathways are involved in NOX5-dependent endothelial cell migration; Figure S7: p38 MAPK, ERK, and JNK pathways are involved in the MMP-10 secretion and endothelial cell migration triggered by Ang II-mediated NOX5 activation.
