# Supplementary material for: NADPH Oxidase 5 (NOX5) Upregulates MMP-10 Production and Cell Migration in Human Endothelial Cells
Source: Antioxidants (Basel). 2024 Oct 3;13(10):1199. doi: 10.3390/antiox13101199 (PMC11504164; doi:10.3390/antiox13101199)
Supplement: Supplementary file 1 [file antioxidants-13-01199-s001.zip › antioxidants-3115826-supplementary.pdf]

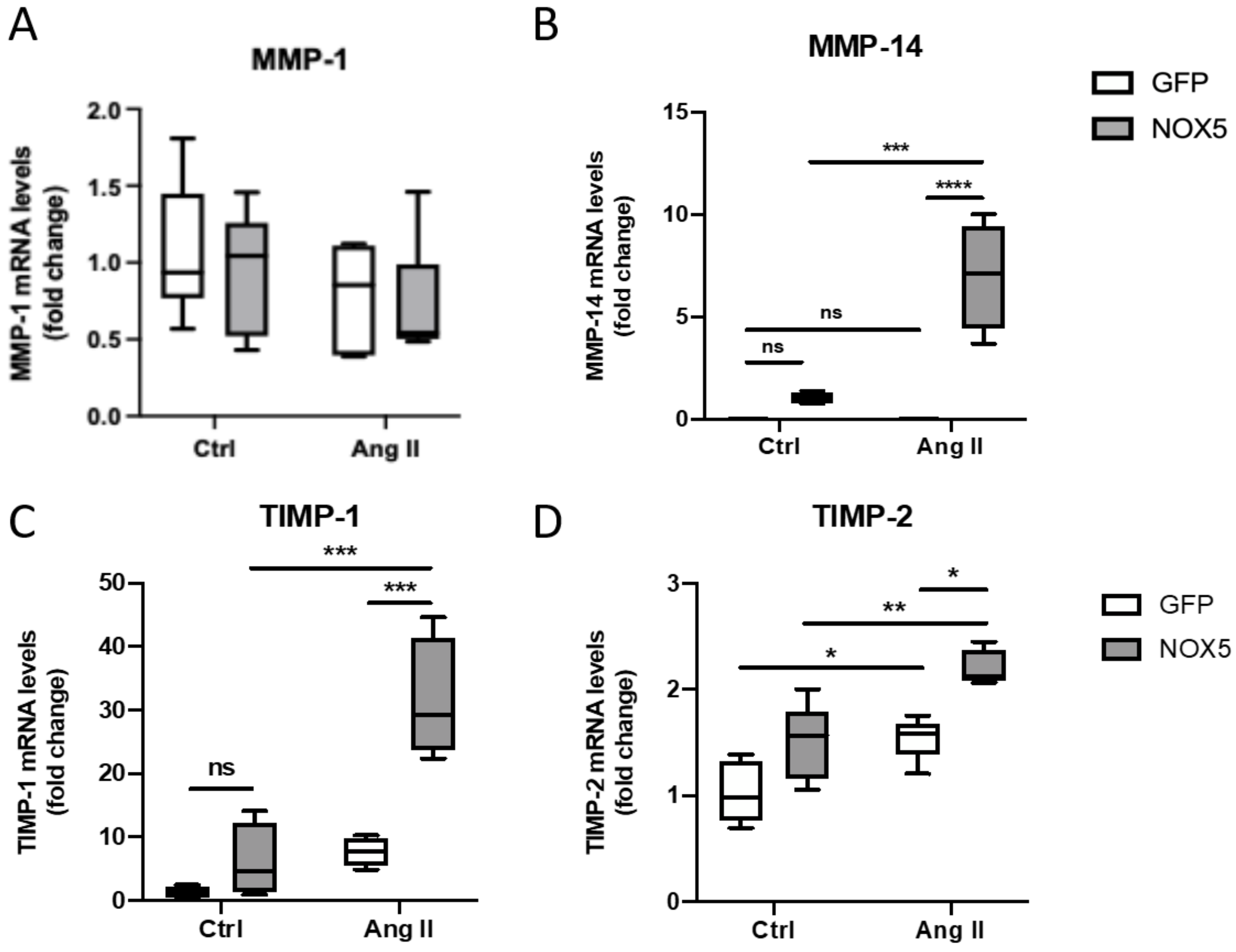

Figure S1. Ang II-mediated NOX5-β stimulation upregulates genes associated with extracellular matrix remodelling. (A) MMP-1 mRNA levels after 24 h of infection and 16 h of Ang II stimulation (n=6). (B) MMP-14 mRNA levels after 24 h of infection and 16 h of Ang II stimulation (n=6). (C) TIMP-1 mRNA levels after 24 h of infection and 16 h of Ang II stimulation (n=6). (D) TIMP-2 mRNA levels after 24 h of infection and 16 h of Ang II stimulation (n=6). GFP: teloHAEC infected with GFP-encoding adenovirus. NOX5: teloHAEC infected with NOX5-encoding adenovirus. Ctrl: non-stimulated cells. Ang II: cells stimulated with Ang II 0.1 μM. ns: not significant differences, *$p<0.05$, **$p<0.01$, ***$p<0.001$, ****$p<0.0001$. Data are presented as median and IQR.

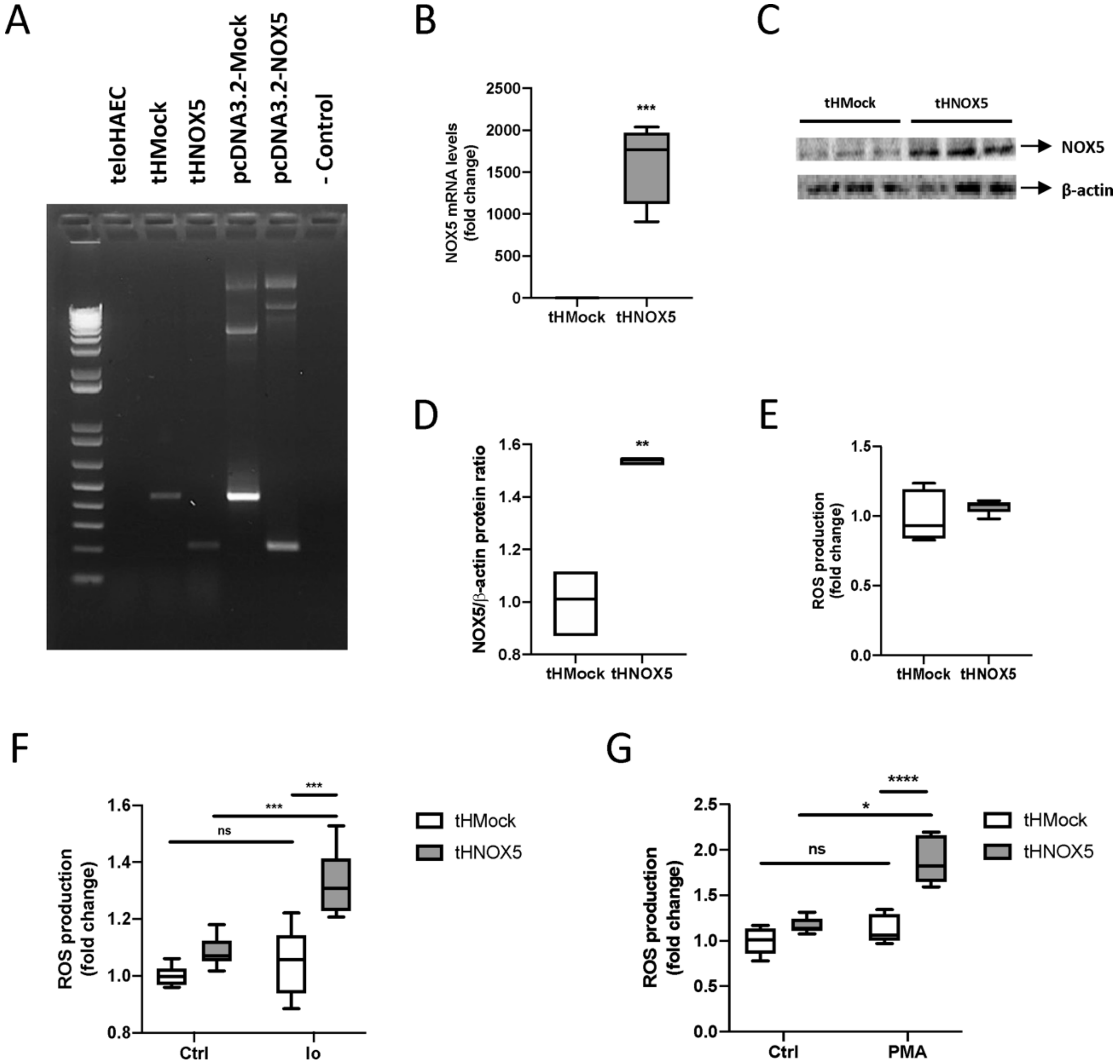

Figure S2. The stable teloHAEC cell line expressing NOX5 (tHNOX5) serves as model of chronic NOX5 overexpression. (A) Agarose gel image of the genotyping PCR. Specific primers P1, P2 and P3 were used to detect Mock and NOX5 plasmid sequences in the gDNA of teloHAEC cell lines. Lane 1: molecular weight marker. Lane 2: teloHAEC gDNA amplification. Lane 3: tHMock gDNA PCR amplification. Lane 4: tHNOX5 gDNA PCR amplification. Lane 5: pcDNA3.2-Mock plasmid PCR amplification. Lane 6: pcDNA3.2-NOX5 plasmid PCR amplification. Lane 7: PCR negative control. (B) NOX5 mRNA levels of tHMock and tHNOX5 cell lines (n=6). (C) NOX5 and β-actin immunoblots of tHMock and tHNOX5 cells (n=3). (D) NOX5 protein levels quantification of tHMock and tHNOX5 cells (n=3). (E-G): ROS leves produced by tHMock and tHNOX5 cell lines at baseline (E; n=6) and after Io (F; n=6) or PMA (G; n=6) stimulation. tHMock: stable cell line transfected with pcDNA3.2-Mock. tHNOX5: stable cell line transfected with pcDNA3.2-NOX5. ns: not significative differences, *$p<0.05$, **$p<0.01$, ***$p<0.001$, ****$p<0.0001$. Data are presented as median and IQR. Io: 100 nM ionomycine; PMA: 100 nM phorbol 12-myristate 13-acetate.

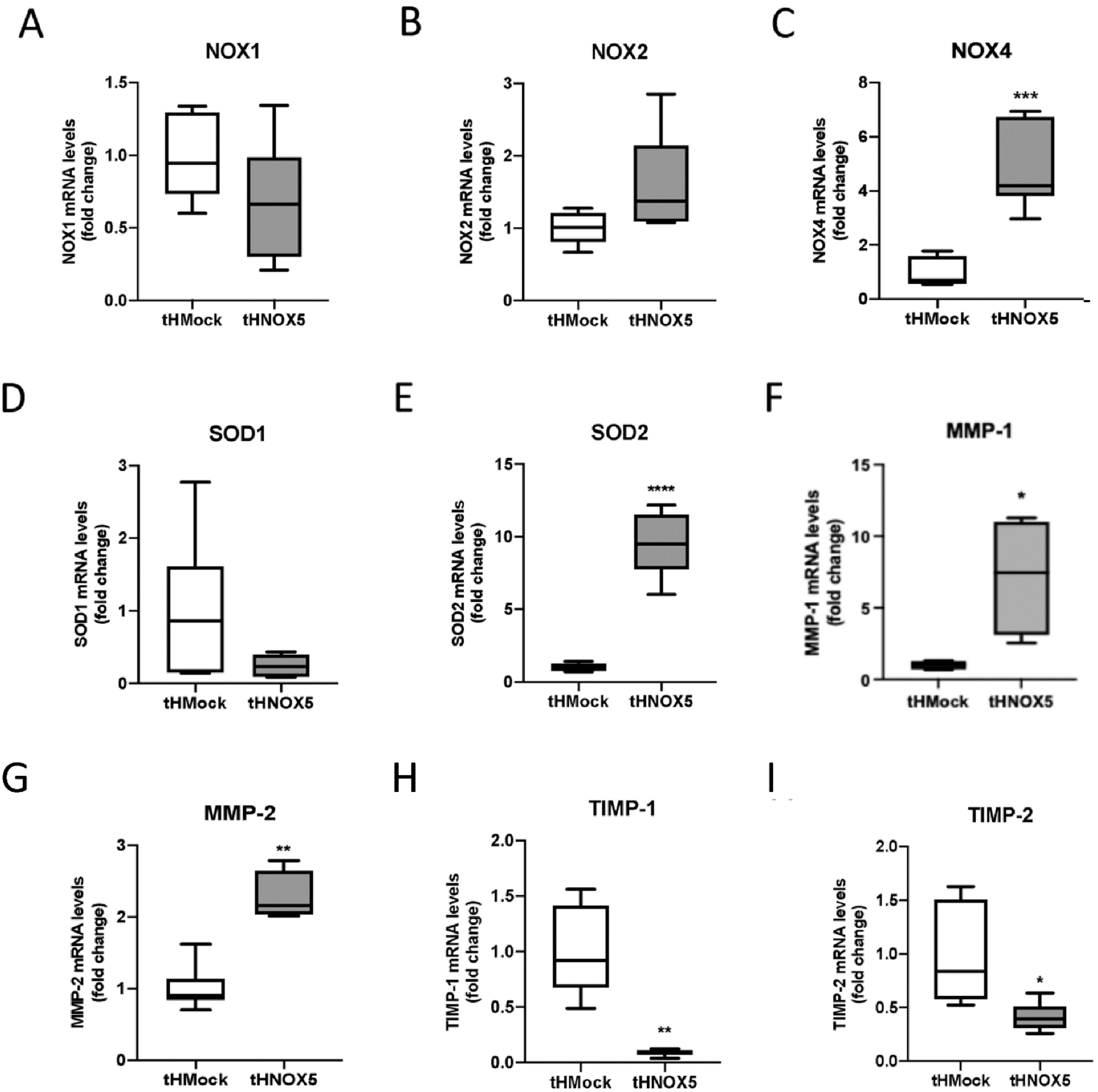

Figure S3. NOX5 chronic overexpression affects other redox and MMP-related genes. In tHMock and tHNOX5 cell lines mRNA levels of NOX1 (A), NOX2 (B), NOX4 (C), SOD1 (D), SOD2 (E), MMP-1 (F), MMP-2 (G), TIMP-1 (H) and TIMP-2 (I) were analyzed by real time qPCR (n=6). tHMock: stable cell line transfected with pcDNA3.2-Mock. tHNOX5: stable cell line transfected with pcDNA3.2-NOX5. *p<0.05, **p<0.01, ***p<0.001, ****p<0.0001. Data are presented as median and IQR.

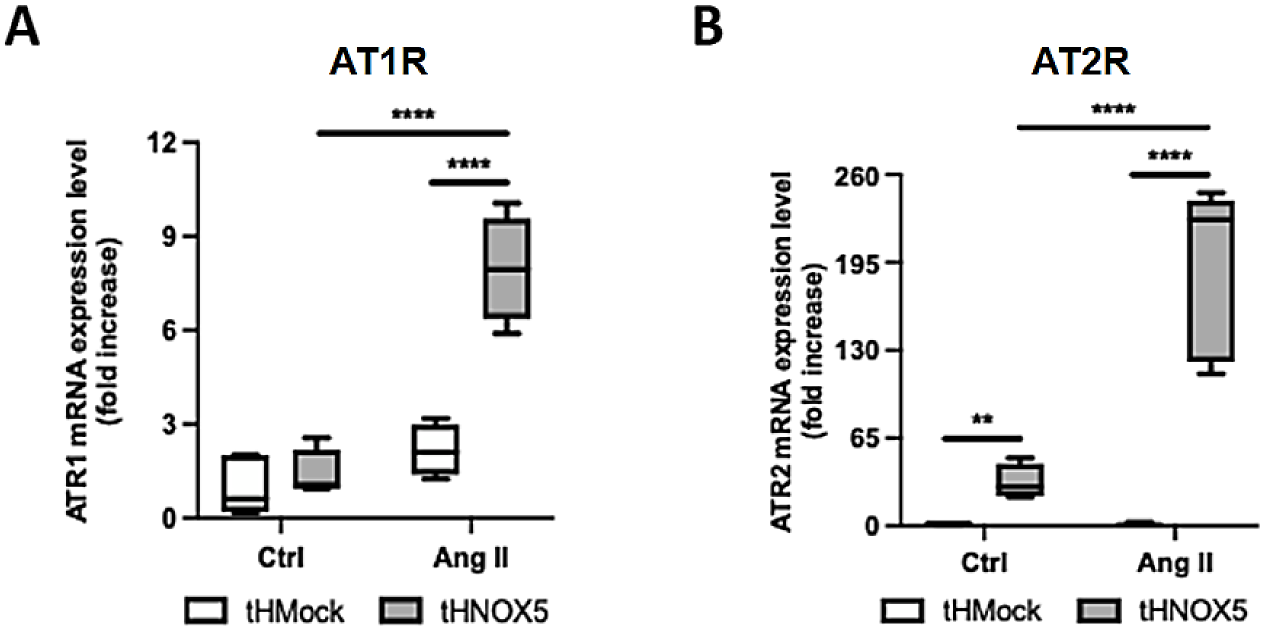

Figure S4. NOX5 chronic overexpression seems to affect Ang II receptors (AT1R and AT2R) expression, with a more intense effect on AT2R. (A) AT1R mRNA levels of tHMock and tHNOX5 cell lines after 8 h of Ang II stimulation (n=4). (B) AT2R mRNA levels of tHMock and tHNOX5 cell lines after 8 h of Ang II stimulation (n=4). tHMock: stable cell line transfected with pcDNA3.2-Mock. tHNOX5: stable cell line transfected with pcDNA3.2-NOX5. **p<0.01, ****p<0.0001. Data are presented as median and IQR.

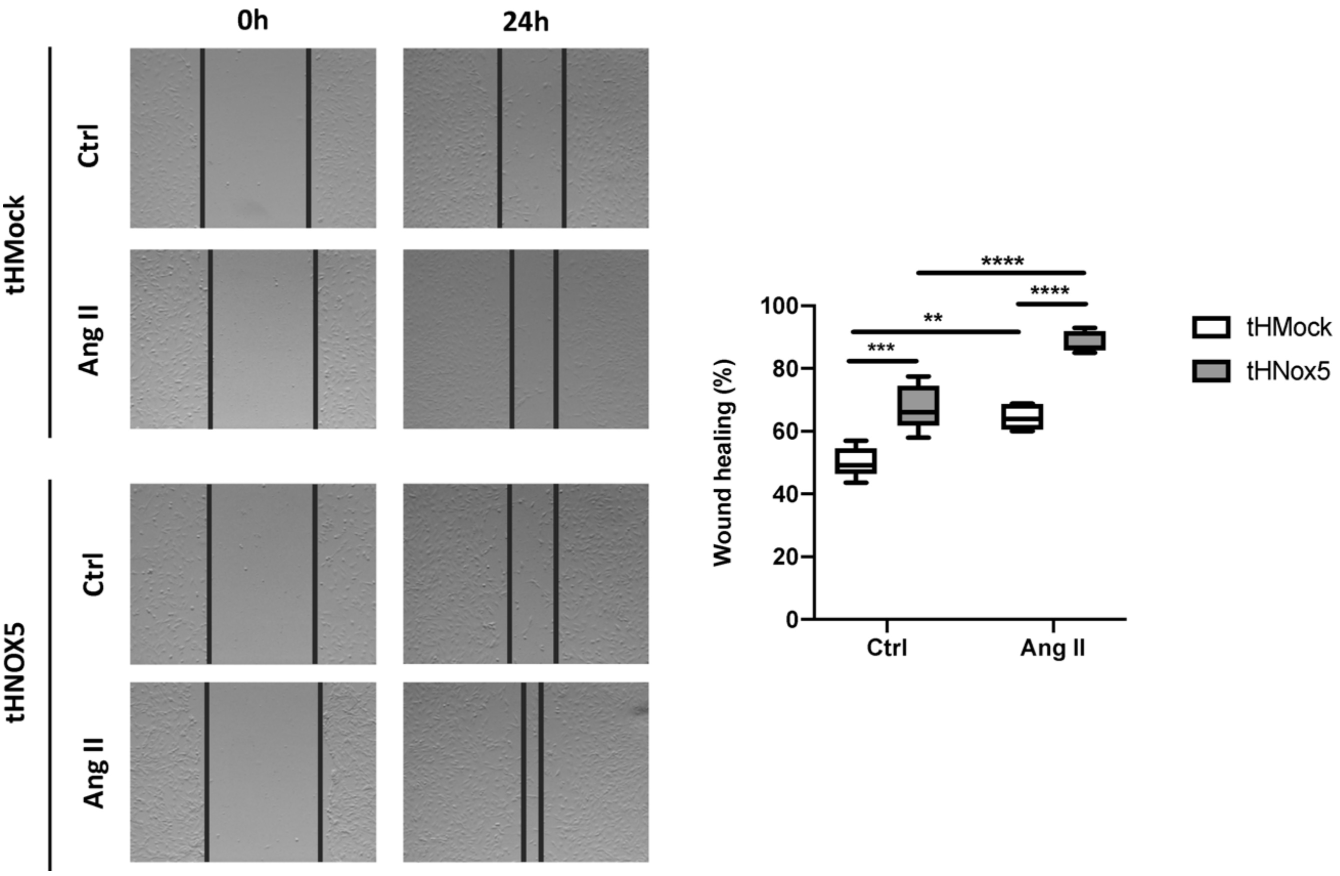

Figure S5. Ang II potentiates the promigratory phenotype of NOX5 overexpressing cells. Left panel: Representative images of tHMock and tHNOX5 cells at baseline and stimulated with 0.25μM Ang II 0 and 24 h after the scratch of wound healing assay. Right panel: Quantification of the wound healing assay of tHMock and tHNOX5 cultures at baseline and stimulated with 0.25 μM Ang II (n=6). tHMock: stable cell line transfected with pcDNA3.2-Mock. tHNOX5: stable cell line transfected with pcDNA3.2-NOX5. ns: not significant differences, **p<0.01, ***p<0.001, ****p<0.0001. Data are presented as median and IQR.

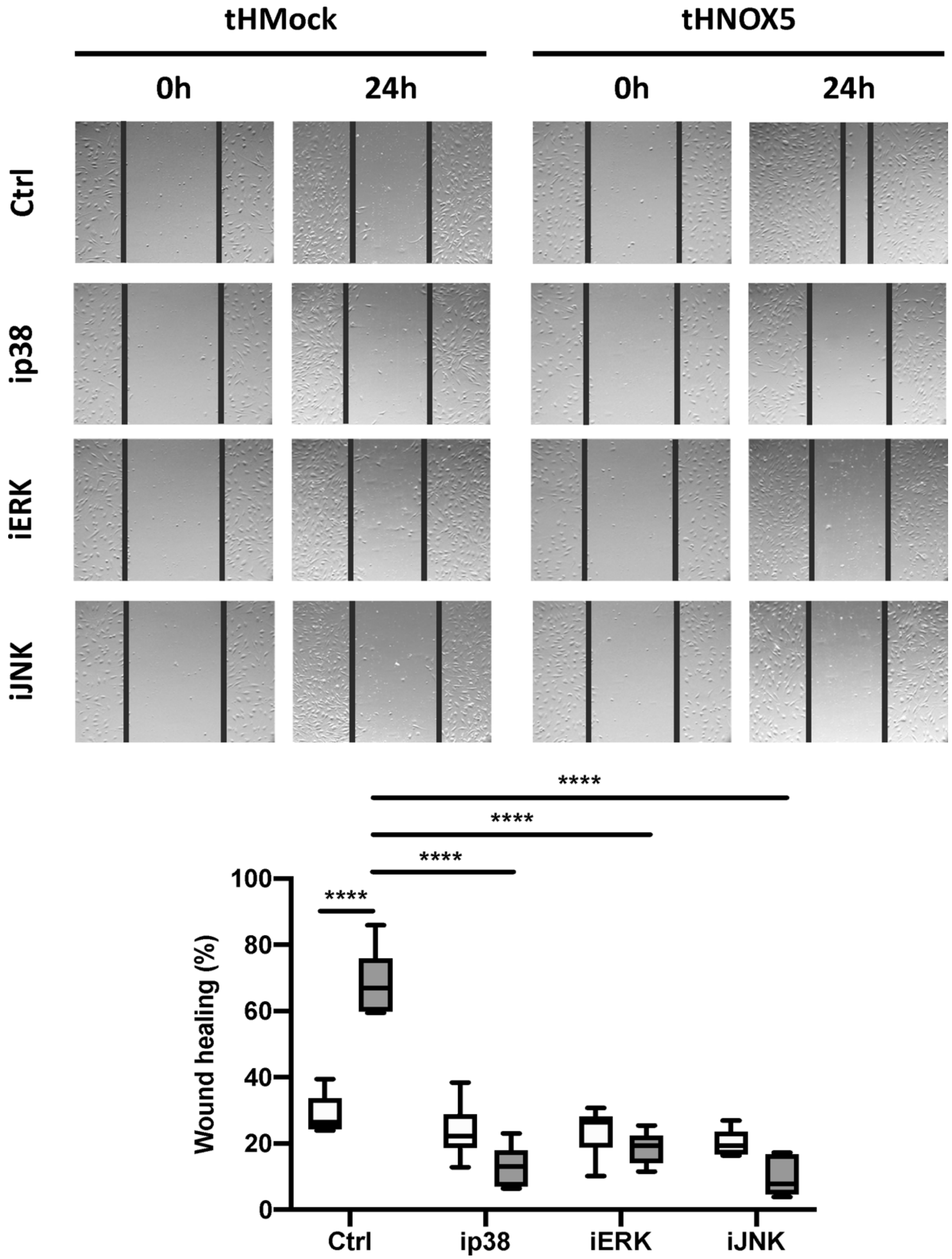

Figure S6. p38 MAPK, MEK/ERK and JNK pathways are involved NOX5-dependent endothelial cell migration. Upper panel: Representative images of tHMock and tHNOX5 cells in the absence (Ctrl) or presence of 5 μM 219138-24-6 (p28 MAPK inhibitor, ip38), PD98059 (MEK/ERK inhibitor, iERK) or JNK-IN-8 (JNK inhibitor, iJNK) 0 and 24 h after the scratch of wound healing assay (n=6). Bottom panel: Quantification of the wound healing assay. tHMock: stable cell line transfected with pcDNA3.2-Mock. tHNOX5: stable cell line transfected with pcDNA3.2-NOX5. ****p<0.0001. Data are presented as median and IQR.

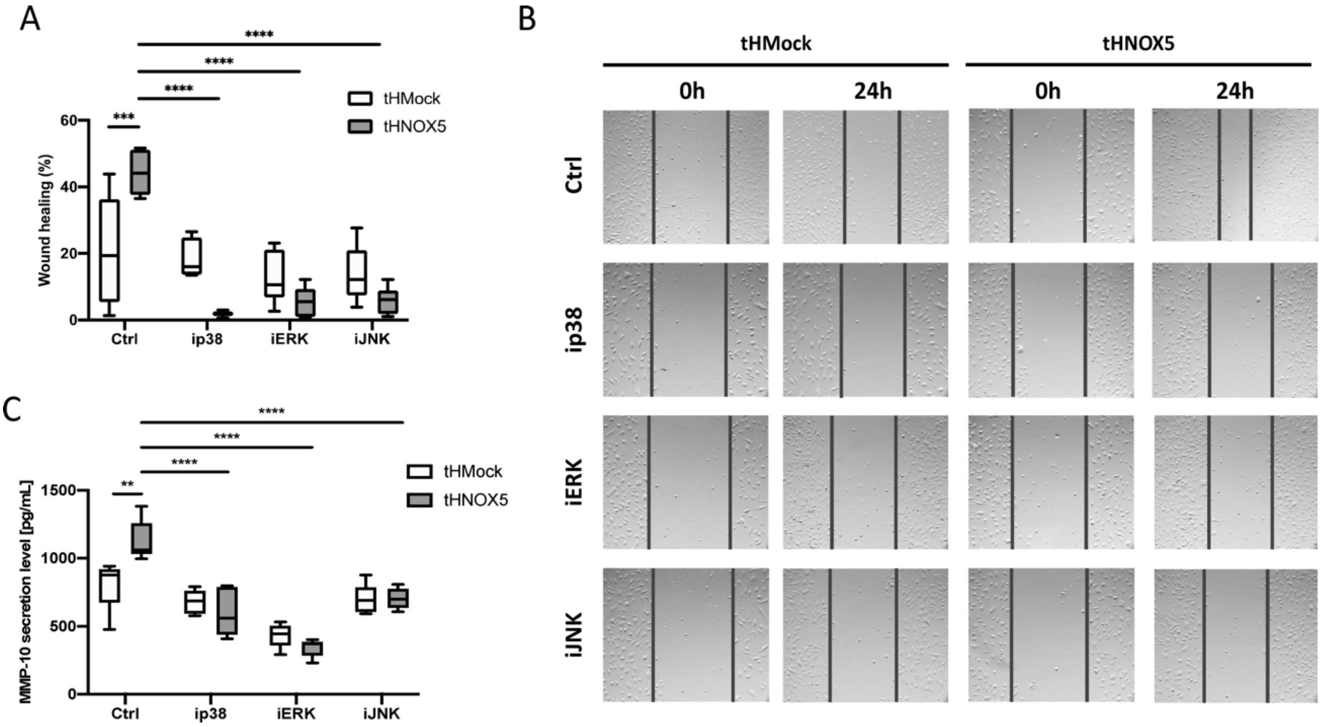

Figure S7. p38 MAPK, ERK and JNK pathways are involved in the MMP-10 secretion and endothelial cell migration triggered by Ang II-mediated NOX5 activation. (A) Quantification of the wound healing assay of tHMock and tHNOX5 control cells in the absence or presence of 5 μM 219138-24-6 (p28 MAPK inhibitor, ip38), PD98059 (MEK/ERK inhibitor, iERK) or JNK-IN-8 (JNK inhibitor, iJNK) 0 and 24 h after the scratch of wound healing assay and Ang II stimulation (n=6). (B) Representative images of the wound healing assay. (C) Accumulated MMP-10 protein in cell supernatant from tHMock and tHNOX5 cells treated as indicated in A and stimulated with Ang II for 48 h (n=6). tHMock: stable cell line transfected with pcDNA3.2-Mock. tHNOX5: stable cell line transfected with pcDNA3.2-NOX5. **p<0.01, ***p<0.001, ****p<0.0001. Data are presented as median and IQR.

pcDNA3.2-NOX5β expression plasmid (7,642 nucleotides)

5’GACGGATCGGGAGATCTCCCGATCCCCTATGGTCGACTCTCAGTACAATCTGCTCTGATGCCGCATAGTTAAG
CCAGTATCTGCTCCCTGCTTGTGTGTTGGAGGTCGCTGAGTAGTGCGCGAGCAAAATTTAAGCTACAACAAGG
CAAGGCTTGACCGACAATTGCATGAAGAATCTGCTTAGGGTTAGGCGTTTTGCGCTGCTTCGCGATGTACGGG
CCAGATATACGCGTTGACATTGATTATTGACTAGTTATTAATAGTAATCAATTACGGGGTCATTAGTTCATAGCCCA
TATATGGAGTTCCGCGTTACATAACTTACGGTAAATGGCCCGCCTGGCTGACCGCCCAACGACCCCCGCCCATT
GACGTCAATAATGACGTATGTTCCCATAGTAACGCCAATAGGGACTTTCCATTGACGTCAATGGGTGGACTATTT
ACGGTAAACTGCCCACTTGGCAGTACATCAAGTGTATCATATGCCAAGTACGCCCCCTATTGACGTCAATGACG
GTAAATGGCCCGCCTGGCATTATGCCCAGTACATGACCTTATGGGACTTTCCTACTTGGCAGTACATCTACGTAT
TAGTCATCGCTATTACCATGGTGATGCGGTTTTGGCAGTACATCAATGGGCGTGGATAGCGGTTTGACTCACGG
GGATTTCCAAGTCTCCACCCCATTGACGTCAATGGGAGTTTGTTTTGGCACCAAAATCAACGGGACTTTCCAAA
ATGTCGTAACAACTCCGCCCCATTGACGCAAATGGGCGGTAGGCGTGTACGGTGGGAGGTCTATATAAGCAGA
GCTCTCTGGCTAACTAGAGAACCCACTGCTTACTGGCTTATCGAAATTAATACGACTCACTATAGGGAGACCCAA
GCTGGCTAGTTAAGCTATCAACAAGTTTGTACAAACACC**ATG**AGTGCCGAGGAGGATGCCAGGTGGCTCCGGT
GGGTGACTCAGCAGTTTAAGACCATTGCAGGAGAAGATGGGGAGATCAGCCTGCAAGAATTCAAAGCAGCTCT
GCATGTGAAAGAGTCCTTCTTTGCAGAGCGATTCTTTGCCCTATTTGACTCCGATAGAAGTGGCACCATCACCC
TCCAGGAGCTGCAGGAGGCACTGACCCTGCTCATCCATGGCAGCCCCATGGACAAACTCAAATTCCTCTTCCA
GGTGTATGACATCGATGGCAGTGGCTCCATTGACCCGGATGAGCTGCGCACTGTGCTGCAGTCGTGTCTGCG
CGAGAGCGCCATCTCGCTGCCTGACGAGAAGCTGGACCAGCTGACGCTGGCGCTCTTCGAATCGGCCGACG
CGGACGGCAACGGGGCCATCACCTTCGAGGAGCTCCGGGACGAGCTGCAGCGCTTCCCCGGAGTCATGGAG
AACCTGACCATCAGCGCTGCCCACTGGCTGACGGCCCCCGCCCCCCGCCCACGCCCGCGCCGGCCGCGCC
AGCTGACCCGCGCCTACTGGCACAACCACCGCAGCCAGCTGTTCTGCCTGGCCACCTATGCAGGCCTCCACG
TGCTGCTCTTCGGGCTGGCGGCCAGCGCGCACCGGGACCTCGGCGCCAGCGTCATGGTGGCCAAGGGCTG
TGGCCAGTGCCTCAACTTCGACTGCAGCTTCATCGCGGTGCTGATGCTCAGACGCTGCCTCACCTGGCTGCG
GGCCACGTGGCTGGCTCAAGTCCTACCACTGGACCAGAACATCCAGTTCCACCAGCTTATGGGCTACGTGGTA
GTGGGGCTGTCCCTCGTGCACACTGTGGCTCACACTGTGAACTTTGTACTCCAGGCTCAGGCGGAGGCCAGC
CCTTTCCAGTTCTGGGAGCTGCTGCTCACCACGAGGCCTGGCATTGGCTGGGTACACGGTTCGGCCTCCCCG
ACAGGTGTCGCTCTGCTGCTGCTGCTCCTCCTCATGTTCATCTGCTCCAGTTCCTGCATCCGCAGGAGTGGCC
ACTTTGAGGTGTTCTATTGGACTCACCTGTCCTACCTCCTCGTGTGGCTTCTGCTCATCTTTCATGGGCCCAAC
TTCTGGAAGTGGCTGCTGGTGCCTGGAATCTTGTTTTTCCTGGAGAAGGCCATCGGACTGGCAGTGTCCCGCA
TGGCAGCCGTGTGCATCATGGAAGTCAACCTCCTCCCCTCCAAGGTCACTCATCTCCTCATCAAGCGGCCCCC
TTTTTTTCACTATAGACCTGGTGACTACTTGTATCTGAACATCCCCACCATTGCTCGCTATGAGTGGCACCCCTT
CACCATCAGCAGTGCTCCTGAGCAGAAAGACACTATCTGGCTGCACATTCGGTCCCAAGGCCAGTGGACAAAC
AGGCTGTATGAGTCCTTCAAGGCATCAGACCCACTGGGCCGTGGTTCTAAGAGGCTGTCGAGGAGTGTGACA
ATGAGAAAGAGTCAAAGGTCGTCCAAGGGCTCTGAGATACTTTTGGAGAAACACAAATTCTGTAACATCAAGTG
CTACATCGATGGGCCTTATGGGACCCCCACCCGCAGGATCTTTGCCTCTGAGCATGCCGTGCTCATCGGGGCA
GGCATCGGCATCACCCCCTTTGCTTCCATTCTGCAGAGTATCATGTACAGGCACCAGAAAAGAAAGCATACTTG
CCCCAGCTGCCAGCACTCCTGGATCGAAGGTGTCCAAGACAACATGAAGCTCCATAAGGTGGACTTTATCTGG
ATCAACAGAGACCAGCGGTCTTTCGAGTGGTTTGTGAGCCTGCTGACTAAACTGGAGATGGACCAGGCCGAG
GAGGCTCAATACGGCCGCTTCCTGGAGCTGCATATGTACATGACATCTGCACTGGGCAAGAATGACATGAAGG
CCATTGGCCTGCAGATGGCCCTTGACCTCCTGGCCAACAAGGAGAAGAAAGACTCCATCACGGGGCTGCAGA
CGCGCACCCAGCCTGGGCGGCCTGACTGGAGCAAGGTGTTCCAGAAAGTGGCTGCTGAGAAGAAGGGCAAG
GTGCAGGTCTTCTTCTGTGGCTCCCCAGCTCTGGCCAAGGTGCTGAAGGGCCATTGTGAGAAGTTCGGCTTC
AGATTTTTCCAAGAGAATTTC**TAG**CTCGAGTACAAAGTGGTTGATCTAGAGGGCCCGCGGTTCGAAGGTAAGCC
TATCCCTAACCCTCTCCTCGGTCTCGATTCTACGCGTACCGGTTAGTAATGAGTTTAAACGGGGGAGGCTAACT
GAAACACGGAAGGAGACAATACCGGAAGGAACCCGCGCTATGACGGCAATAAAAAGACAGAATAAAACGCACG
GGTGTTGGGTCGTTTGTTCATAAACGCGGGGTTCGGTCCCAGGGCTGGCACTCTGTCGATACCCCACCGAGA
CCCCATTGGGGCCAATACGCCCGCGTTTCTTCCTTTTCCCCACCCCACCCCCCAAGTTCGGGTGAAGGCCCA
GGGCTCGCAGCCAACGTCGGGGCGGCAGGCCCTGCCATAGCAGATCTGCGCAGCTGGGGCTCTAGGGGGTA
TCCCCACGCGCCCTGTAGCGGCGCATTAAGCGCGGCGGGTGTGGTGGTTACGCGCAGCGTGACCGCTACACT
TGCCAGCGCCCTAGCGCCCGCTCCTTTCGCTTTCTTCCCTTCCTTTCTCGCCACGTTCGCCGGCTTTCCCCGT
CAAGCTCTAAATCGGGGCATCCCTTTAGGGTTCCGATTTAGTGCTTTACGGCACCTCGACCCCAAAAAACTTGA
TTAGGGTGATGGTTCACGTAGTGGGCCATCGCCCTGATAGACGGTTTTTCGCCCTTTGACGTTGGAGTCCACG
TTCTTTAATAGTGGACTCTTGTTCCAAACTGGAACAACACTCAACCCTATCTCGGTCTATTCTTTTGATTTATAAG
GGATTTTGGGGATTTCGGCCTATTGGTTAAAAAATGAGCTGATTTAACAAAAATTTAACGCGAATTAATTCTGTGG
AATGTGTGTCAGTTAGGGTGTGGAAAGTCCCCAGGCTCCCCAGCAGGCAGAAGTATGCAAAGCATGCATCTCA
ATTAGTCAGCAACCAGGTGTGGAAAGTCCCCAGGCTCCCCAGCAGGCAGAAGTATGCAAAGCATGCATCTCAA
TTAGTCAGCAACCATAGTCCCGCCCCTAACTCCGCCCATCCCGCCCCTAACTCCGCCCAGTTCCGCCCATTCT
CCGCCCCATGGCTGACTAATTTTTTTTATTTATGCAGAGGCCGAGGCCGCCTCTGCCTCTGAGCTATTCCAGAA

GTAGTGAGGAGGCTTTTTTGGAGGCCTAGGCTTTTGCAAAAAGCTCCCGGGAGCTTGTATATCCATTTTCGGAT
CTGATCAAGAGACAGGATGAGGATCGTTTCGCATGATTGAACAAGATGGATTGCACGCAGGTTCTCCGGCCGC
TTGGGTGGAGAGGCTATTCGGCTATGACTGGGCACAACAGACAATCGGCTGCTCTGATGCCGCCGTGTTCCG
GCTGTCAGCGCAGGGGCGCCCGGTTCTTTTTGTCAAGACCGACCTGTCCGGTGCCCTGAATGAACTGCAGGA
CGAGGCAGCGCGGCTATCGTGGCTGGCCACGACGGGCGTTCCTTGCGCAGCTGTGCTCGACGTTGTCACTG
AAGCGGGAAGGGACTGGCTGCTATTGGGCGAAGTGCCGGGGCAGGATCTCCTGTCATCTCACCTTGCTCCTG
CCGAGAAAGTATCCATCATGGCTGATGCAATGCGGCGGCTGCATACGCTTGATCCGGCTACCTGCCCATTCGAC
CACCAAGCGAAACATCGCATCGAGCGAGCACGTACTCGGATGGAAGCCGGTCTTGTCGATCAGGATGATCTGG
ACGAAGAGCATCAGGGGCTCGCGCCAGCCGAACTGTTCGCCAGGCTCAAGGCGCGCATGCCCGACGGCGAG
GATCTCGTCGTGACCCATGGCGATGCCTGCTTGCCGAATATCATGGTGGAAAATGGCCGCTTTTCTGGATTCAT
CGACTGTGGCCGGCTGGGTGTGGCGGACCGCTATCAGGACATAGCGTTGGCTACCCGTGATATTGCTGAAGA
GCTTGGCGGCGAATGGGCTGACCGCTTCCTCGTGCTTTACGGTATCGCCGCTCCCGATTCGCAGCGCATCGC
CTTCTATCGCCTTCTTGACGAGTTCTTCTGAGCGGGACTCTGGGGTTCGCGAAATGACCGACCAAGCGACGCC
CAACCTGCCATCACGAGATTTCGATTCCACCGCCGCCTTCTATGAAAGGTTGGGCTTCGGAATCGTTTTCCGGG
ACGCCGGCTGGATGATCCTCCAGCGCGGGGATCTCATGCTGGAGTTCTTCGCCCACCCCAACTTGTTTATTGC
AGCTTATAATGGTTACAAATAAAGCAATAGCATCACAAATTTCACAAATAAAGCATTTTTTTCACTGCATTCTAGTT
GTGGTTTGTCCAAACTCATCAATGTATCTTATCATGTCTGTATACCGTCGACCTCTAGCTAGAGCTTGGCGTAATC
ATGGTCATAGCTGTTTCCTGTGTGAAATTGTTATCCGCTCACAATTCCACACAACATACGAGCCGGAAGCATAAA
GTGTAAAGCCTGGGGTGCCTAATGAGTGAGCTAACTCACATTAATTGCGTTGCGCTCACTGCCCGCTTTCCAGT
CGGGAAACCTGTCGTGCCAGCTGCATTAATGAATCGGCCAACGCGCGGGGAGAGGCGGTTTGCGTATTGGGC
GCTCTTCCGCTTCCTCGCTCACTGACTCGCTGCGCTCGGTCGTTCGGCTGCGGCGAGCGGTATCAGCTCACT
CAAAGGCGGTAATACGGTTATCCACAGAATCAGGGGATAACGCAGGAAAGAACATGTGAGCAAAAGGCCAGCA
AAAGGCCAGGAACCGTAAAAAGGCCGCGTTGCTGGCGTTTTTCCATAGGCTCCGCCCCCCTGACGAGCATCA
CAAAAATCGACGCTCAAGTCAGAGGTGGCGAAACCCGACAGGACTATAAAGATACCAGGCGTTTCCCCCTGGA
AGCTCCCTCGTGCGCTCTCCTGTTCCGACCCTGCCGCTTACCGGATACCTGTCCGCCTTTCTCCCTTCGGGAA
GCGTGGCGCTTTCTCAATGCTCACGCTGTAGGTATCTCAGTTCGGTGTAGGTCGTTCGCTCCAAGCTGGGCTG
TGTGCACGAACCCCCCGTTCAGCCCGACCGCTGCGCCTTATCCGGTAACTATCGTCTTGAGTCCAACCCGGTA
AGACACGACTTATCGCCACTGGCAGCAGCCACTGGTAACAGGATTAGCAGAGCGAGGTATGTAGGCGGTGCTA
CAGAGTTCTTGAAGTGGTGGCCTAACTACGGCTACACTAGAAGGACAGTATTTGGTATCTGCGCTCTGCTGAAG
CCAGTTACCTTCGGAAAAAGAGTTGGTAGCTCTTGATCCGGCAAACAAACCACCGCTGGTAGCGGTGGTTTTTT
TGTTTGCAAGCAGCAGATTACGCGCAGAAAAAAAGGATCTCAAGAAGATCCTTTGATCTTTTCTACGGGGTCTG
ACGCTCAGTGGAACGAAAACTCACGTTAAGGGATTTTGGTCATGAGATTATCAAAAAGGATCTTCACCTAGATCC
TTTTAAATTAAAAATGAAGTTTTAAATCAATCTAAAGTATATATGAGTAAACTTGGTCTGACAGTTACCAATGCTTAA
TCAGTGAGGCACCTATCTCAGCGATCTGTCTATTTCGTTCATCCATAGTTGCCTGACTCCCCGTCGTGTAGATAA
CTACGATACGGGAGGGCTTACCATCTGGCCCCAGTGCTGCAATGATACCGCGAGACCCACGCTCACCGGCTCC
AGATTTATCAGCAATAAACCAGCCAGCCGGAAGGGCCGAGCGCAGAAGTGGTCCTGCAACTTTATCCGCCTCC
ATCCAGTCTATTAATTGTTGCCGGGAAGCTAGAGTAAGTAGTTCGCCAGTTAATAGTTTGCGCAACGTTGTTGCC
ATTGCTACAGGCATCGTGGTGTCACGCTCGTCGTTTGGTATGGCTTCATTCAGCTCCGGTTCCCAACGATCAAG
GCGAGTTACATGATCCCCCATGTTGTGCAAAAAAGCGGTTAGCTCCTTCGGTCCTCCGATCGTTGTCAGAAGTA
AGTTGGCCGCAGTGTTATCACTCATGGTTATGGCAGCACTGCATAATTCTCTTACTGTCATGCCATCCGTAAGAT
GCTTTTCTGTGACTGGTGAGTACTCAACCAAGTCATTCTGAGAATAGTGTATGCGGCGACCGAGTTGCTCTTGC
CCGGCGTCAATACGGGATAATACCGCGCCACATAGCAGAACTTTAAAAGTGCTCATCATTGGAAAACGTTCTTC
GGGGCGAAAACTCTCAAGGATCTTACCGCTGTTGAGATCCAGTTCGATGTAACCCACTCGTGCACCCAACTGAT
CTTCAGCATCTTTTACTTTCACCAGCGTTTCTGGGTGAGCAAAAACAGGAAGGCAAAATGCCGCAAAAAAGGGA
ATAAGGGCGACACGGAAATGTTGAATACTCATACTCTTCCTTTTTCAATATTATTGAAGCATTTATCAGGGTTATTG
TCTCATGAGCGGATACATATTTGAATGTATTTAGAAAAATAAACAAATAGGGGTTCCGCGCACATTTCCCCGAAAA
GTGCCACCTGACGTC 3’

pcDNA3.2 control plasmid (Mock) (5,263 nucleotides)

5’GACGGATCGGGAGATCTCCCGATCCCCTATGGTCGACTCTCAGTACAATCTGCTCTGATGCCGCATAGTTAAG
CCAGTATCTGCTCCCTGCTTGTGTGTTGGAGGTCGCTGAGTAGTGCGCGAGCAAAATTTAAGCTACAACAAGG
CAAGGCTTGACCGACAATTGCATGAAGAATCTGCTTAGGGTTAGGCGTTTTGCGCTGCTTCGCGATGTACGGG
CCAGATATACGCGTTGACATTGATTATTGACTAGTTATTAATAGTAATCAATTACGGGGTCATTAGTTCATAGCCCA
TATATGGAGTTCCGCGTTACATAACTTACGGTAAATGGCCCGCCTGGCTGACCGCCCAACGACCCCCGCCCATT
GACGTCAATAATGACGTATGTTCCCATAGTAACGCCAATAGGGACTTTCCATTGACGTCAATGGGTGGACTATTT

ACGGTAAACTGCCCACTTGGCAGTACATCAAGTGTATCATATGCCAAGTACGCCCCCTATTGACGTCAATGACG
GTAAATGGCCCGCCTGGCATTATGCCCAGTACATGACCTTATGGGACTTTCCTACTTGGCAGTACATCTACGTAT
TAGTCATCGCTATTACCATGGTGATGCGGTTTTGGCAGTACATCAATGGGCGTGGATAGCGGTTTGACTCACGG
GGATTTCCAAGTCTCCACCCCATTGACGTCAATGGGAGTTTGTTTTGGCACCAAAATCAACGGGACTTTCCAAA
ATGTCGTAACAACTCCGCCCCATTGACGCAAATGGGCGGTAGGCGTGTACGGTGGGAGGTCTATATAAGCAGA
GAAACGGGGGAGGCTAACTGAAACACGGAAGGAGACAATACCGGAAGGAACCCGCGCTATGACGGCAATAAA
AAGACAGAATAAAACGCACGGGTGTTGGGTCGTTTGTTCATAAACGCGGGGTTCGGTCCCAGGGCTGGCACT
CTGTCGATACCCCACCGAGACCCCATTGGGGCCAATACGCCCGCGTTTCTTCCTTTTCCCCACCCCACCCCCC
AAGTTCGGGTGAAGGCCCAGGGCTCGCAGCCAACGTCGGGGCGGCAGGCCCTGCCATAGCAGATCTGCGCA
GCTGGGGCTCTAGGGGGTATCCCCACGCGCCCTGTAGCGGCGCATTAAGCGCGGCGGGTGTGGTGGTTACG
CGCAGCGTGACCGCTACACTTGCCAGCGCCCTAGCGCCCGCTCCTTTCGCTTTCTTCCCTTCCTTTCTCGCCA
CGTTCGCCGGCTTTCCCCGTCAAGCTCTAAATCGGGGCATCCCTTTAGGGTTCCGATTTAGTGCTTTACGGCAC
CTCGACCCCAAAAAACTTGATTAGGGTGATGGTTCACGTAGTGGGCCATCGCCCTGATAGACGGTTTTTCGCCC
TTTGACGTTGGAGTCCACGTTCTTTAATAGTGGACTCTTGTTCCAAACTGGAACAACACTCAACCCTATCTCGGT
CTATTCTTTTGATTTATAAGGGATTTTGGGGATTTCGGCCTATTGGTTAAAAAATGAGCTGATTTAACAAAAATTTA
ACGCGAATTAATTCTGTGGAATGTGTGTCAGTTAGGGTGTGGAAAGTCCCCAGGCTCCCCAGCAGGCAGAAGT
ATGCAAAGCATGCATCTCAATTAGTCAGCAACCAGGTGTGGAAAGTCCCCAGGCTCCCCAGCAGGCAGAAGTA
TGCAAAGCATGCATCTCAATTAGTCAGCAACCATAGTCCCGCCCCTAACTCCGCCCATCCCGCCCCTAACTCCG
CCCAGTTCCGCCCATTCTCCGCCCCATGGCTGACTAATTTTTTTTATTTATGCAGAGGCCGAGGCCGCCTCTGC
CTCTGAGCTATTCCAGAAGTAGTGAGGAGGCTTTTTTGGAGGCCTAGGCTTTTGCAAAAAGCTCCCGGGAGCT
TGTATATCCATTTTCGGATCTGATCAAGAGACAGGATGAGGATCGTTTCGCATGATTGAACAAGATGGATTGCAC
GCAGGTTCTCCGGCCGCTTGGGTGGAGAGGCTATTCGGCTATGACTGGGCACAACAGACAATCGGCTGCTCT
GATGCCGCCGTGTTCCGGCTGTCAGCGCAGGGGCGCCCGGTTCTTTTTGTCAAGACCGACCTGTCCGGTGCC
CTGAATGAACTGCAGGACGAGGCAGCGCGGCTATCGTGGCTGGCCACGACGGGCGTTCCTTGCGCAGCTGT
GCTCGACGTTGTCACTGAAGCGGGAAGGGACTGGCTGCTATTGGGCGAAGTGCCGGGGCAGGATCTCCTGTC
ATCTCACCTTGCTCCTGCCGAGAAAGTATCCATCATGGCTGATGCAATGCGGCGGCTGCATACGCTTGATCCGG
CTACCTGCCCATTCGACCACCAAGCGAAACATCGCATCGAGCGAGCACGTACTCGGATGGAAGCCGGTCTTGT
CGATCAGGATGATCTGGACGAAGAGCATCAGGGGCTCGCGCCAGCCGAACTGTTCGCCAGGCTCAAGGCGC
GCATGCCCGACGGCGAGGATCTCGTCGTGACCCATGGCGATGCCTGCTTGCCGAATATCATGGTGGAAAATGG
CCGCTTTTCTGGATTCATCGACTGTGGCCGGCTGGGTGTGGCGGACCGCTATCAGGACATAGCGTTGGCTACC
CGTGATATTGCTGAAGAGCTTGGCGGCGAATGGGCTGACCGCTTCCTCGTGCTTTACGGTATCGCCGCTCCCG
ATTCGCAGCGCATCGCCTTCTATCGCCTTCTTGACGAGTTCTTCTGAGCGGGACTCTGGGGTTCGCGAAATGA
CCGACCAAGCGACGCCCAACCTGCCATCACGAGATTTCGATTCCACCGCCGCCTTCTATGAAAGGTTGGGCTT
CGGAATCGTTTTCCGGGACGCCGGCTGGATGATCCTCCAGCGCGGGGATCTCATGCTGGAGTTCTTCGCCCA
CCCCAACTTGTTTATTGCAGCTTATAATGGTTACAAATAAAGCAATAGCATCACAAATTTCACAAATAAAGCATTTT
TTTCACTGCATTCTAGTTGTGGTTTGTCCAAACTCATCAATGTATCTTATCATGTCTGTATACCGTCGACCTCTAG
CTAGAGCTTGGCGTAATCATGGTCATAGCTGTTTCCTGTGTGAAATTGTTATCCGCTCACAATTCCACACAACATA
CGAGCCGGAAGCATAAAGTGTAAAGCCTGGGGTGCCTAATGAGTGAGCTAACTCACATTAATTGCGTTGCGCTC
ACTGCCCGCTTTCCAGTCGGGAAACCTGTCGTGCCAGCTGCATTAATGAATCGGCCAACGCGCGGGGAGAGG
CGGTTTGCGTATTGGGCGCTCTTCCGCTTCCTCGCTCACTGACTCGCTGCGCTCGGTCGTTCGGCTGCGGCG
AGCGGTATCAGCTCACTCAAAGGCGGTAATACGGTTATCCACAGAATCAGGGGATAACGCAGGAAAGAACATGT
GAGCAAAAGGCCAGCAAAAGGCCAGGAACCGTAAAAAGGCCGCGTTGCTGGCGTTTTTCCATAGGCTCCGCC
CCCCTGACGAGCATCACAAAAATCGACGCTCAAGTCAGAGGTGGCGAAACCCGACAGGACTATAAAGATACCA
GGCGTTTCCCCCTGGAAGCTCCCTCGTGCGCTCTCCTGTTCCGACCCTGCCGCTTACCGGATACCTGTCCGC
CTTTCTCCCTTCGGGAAGCGTGGCGCTTTCTCAATGCTCACGCTGTAGGTATCTCAGTTCGGTGTAGGTCGTTC
GCTCCAAGCTGGGCTGTGTGCACGAACCCCCCGTTCAGCCCGACCGCTGCGCCTTATCCGGTAACTATCGTCT
TGAGTCCAACCCGGTAAGACACGACTTATCGCCACTGGCAGCAGCCACTGGTAACAGGATTAGCAGAGCGAG
GTATGTAGGCGGTGCTACAGAGTTCTTGAAGTGGTGGCCTAACTACGGCTACACTAGAAGGACAGTATTTGGTA
TCTGCGCTCTGCTGAAGCCAGTTACCTTCGGAAAAAGAGTTGGTAGCTCTTGATCCGGCAAACAAACCACCGC
TGGTAGCGGTGGTTTTTTTGTTTGCAAGCAGCAGATTACGCGCAGAAAAAAAGGATCTCAAGAAGATCCTTTGA
TCTTTTCTACGGGGTCTGACGCTCAGTGGAACGAAAACTCACGTTAAGGGATTTTGGTCATGAGATTATCAAAAA
GGATCTTCACCTAGATCCTTTTAAATTAAAAATGAAGTTTTAAATCAATCTAAAGTATATATGAGTAAACTTGGTCTG
ACAGTTACCAATGCTTAATCAGTGAGGCACCTATCTCAGCGATCTGTCTATTTCGTTCATCCATAGTTGCCTGACT
CCCCGTCGTGTAGATAACTACGATACGGGAGGGCTTACCATCTGGCCCCAGTGCTGCAATGATACCGCGAGAC
CCACGCTCACCGGCTCCAGATTTATCAGCAATAAACCAGCCAGCCGGAAGGGCCGAGCGCAGAAGTGGTCCT
GCAACTTTATCCGCCTCCATCCAGTCTATTAATTGTTGCCGGGAAGCTAGAGTAAGTAGTTCGCCAGTTAATAGT
TTGCGCAACGTTGTTGCCATTGCTACAGGCATCGTGGTGTCACGCTCGTCGTTTGGTATGGCTTCATTCAGCTC
CGGTTCCCAACGATCAAGGCGAGTTACATGATCCCCCATGTTGTGCAAAAAAGCGGTTAGCTCCTTCGGTCCTC
CGATCGTTGTCAGAAGTAAGTTGGCCGCAGTGTTATCACTCATGGTTATGGCAGCACTGCATAATTCTCTTACTG
TCATGCCATCCGTAAGATGCTTTTCTGTGACTGGTGAGTACTCAACCAAGTCATTCTGAGAATAGTGTATGCGGC

GACCGAGTTGCTCTTGCCCGGCGTCAATACGGGATAATACCGCGCCACATAGCAGAACTTTAAAAGTGCTCATC
ATTGGAAAACGTTCTTCGGGGCGAAAACTCTCAAGGATCTTACCGCTGTTGAGATCCAGTTCGATGTAACCCAC
TCGTGCACCCAACTGATCTTCAGCATCTTTTACTTTCACCAGCGTTTCTGGGTGAGCAAAAACAGGAAGGCAAA
ATGCCGCAAAAAAGGGAATAAGGGCGACACGGAAATGTTGAATACTCATACTCTTCCTTTTTCAATATTATTGAAG
CATTTATCAGGGTTATTGTCTCATGAGCGGATACATATTTGAATGTATTTAGAAAAATAAACAAATAGGGGTTCCGC
GCACATTTCCCCGAAAAGTGCCACCTGACGTC 3’
